# Trajectories from single-cells to PAX5-driven leukemia reveal PAX5-MYC interplay in vivo

**DOI:** 10.1038/s41375-025-02626-2

**Published:** 2025-05-20

**Authors:** Franziska Auer, Mina N. F. Morcos, Mikko Sipola, Irfan Akhtar, Sanni Moisio, Julia Vogt, Rebecca Haag, Mari Lahnalampi, Tiina J. Tuononen, Andrea Hanel, Anna Viitasalo, Ulrike A. Friedrich, Andreas Dahl, Carolin Prexler, Aleksandra A. Pandyra, Polina Stepensky, Masatoshi Takagi, Arndt Borkhardt, Merja Heinäniemi, Julia Hauer

**Affiliations:** 1 Technical University of Munich, Germany; School of Medicine and Health; Department of Pediatrics, Munich, Germany; 2 Institute of Biomedicine, School of Medicine, University of Eastern Finland, Yliopistonranta 8, FI-70211 Kuopio, Finland; 3 DRESDEN-concept Genome Center, Technology Platform at the Center for Molecular and Cellular Bioengineering (CMCB), Dresden University of Technology (TUD), Dresden, Germany; 4 German Center for Diabetes Research (DZD e.V.), 85764 Neuherberg, Germany; 5 Paul Langerhans Institute Dresden of the Helmholtz Center Munich, University Hospital and Faculty of Medicine Carl Gustav Carus, Dresden University of Technology (TUD), Dresden, Germany; 6 Institute of Clinical Chemistry and Clinical Pharmacology, University Hospital Bonn, Venusberg-Campus 1, 53127 Bonn, Germany; 7 German Center for Infection Research (DZIF), Partner Site Bonn-Cologne, Bonn, Germany; 8 Department of Pediatric Oncology, Hematology and Clinical Immunology, Heinrich-Heine University Duesseldorf, Medical Faculty, Duesseldorf, Germany; 9 Department of Bone Marrow Transplantation and Cancer Immunotherapy, Hadassah Medical Center, Faculty of Medicine, Hebrew University of Jerusalem, Jerusalem, Israel; 10 Department of Pediatrics and Developmental Biology, Institute of Science Tokyo, Tokyo, Japan; 11 German Cancer Consortium (DKTK), München, Germany; 12 Pediatric Hematology and Oncology, Department of Pediatrics, University Hospital Carl Gustav Carus, Technical University of Dresden, Dresden, Germany; 13 German Center for Child and Adolescent Health (DZKJ), partner site Munich, Munich, Germany

**Keywords:** Acute lymphocytic leukaemia, Cancer models, Risk factors, Cell signalling, B-cell receptor

## Abstract

PAX5 acts as a master regulator of B-cell proliferation and differentiation. Its germline and somatic deregulation have both been implicated in the development of B-cell precursor acute lymphoblastic leukemia (BCP-ALL). However, the process how reduced *PAX5* transcriptional activity mediates progression to BCP-ALL, is still poorly understood. Here, we characterized the longitudinal effects of PAX5 reduction on healthy, pre-leukemic and BCP-ALL cells at the single-cell level. Cell-surface marker analysis revealed a genotype-driven enrichment of the pre-BII population in healthy *Pax5*^±^ mice. This population showed downregulated B-cell receptor signaling, while DNA replication/repair and cell-cycle signaling pathways were upregulated. Moreover, we observed a shift in the kappa/lambda light chain ratio toward lambda rearranged B-cells. Transplantation experiments further validated a delay of *Pax5*^±^ pre-BII cells in maturation and transition to IgM-positivity. Additionally, single-cell RNA-Sequencing and bulk ATAC-Sequencing of different stages of BCP-ALL evolution showed that *Pax5*^±^ pre-leukemic cells lose their B-cell identity and display *Myc* activation. Subsequently, BCP-ALLs acquired additional RAG-mediated aberrations and driver mutations in JAK-STAT and RAS-signaling pathways. Together, this study elucidates molecular and functional checkpoints in PAX5-mediated pre-leukemic cell progression exploitable for therapeutic intervention and demonstrates that PAX5 reduction is sufficient to initiate clonal evolution to BCP-ALL through activation of MYC.

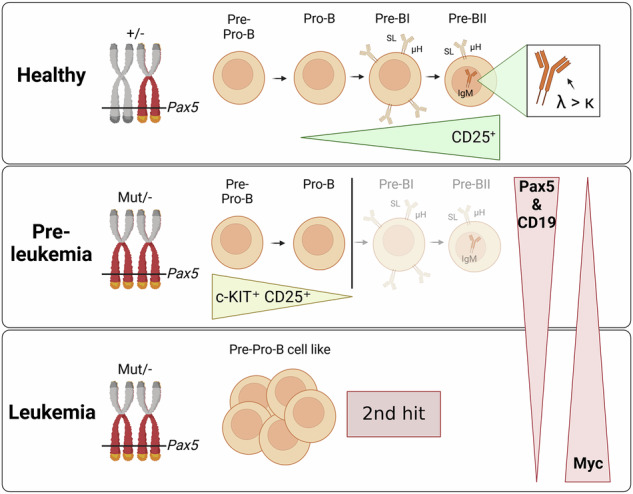

## Introduction

PAX5 is a well-known master regulator for B-cell development, commitment, and identity, exerting functions as a transcriptional activator of B-cell lineage genes, while at the same time repressing lineage-inappropriate genes [1, 2]. By inducing and eliminating active chromatin [3], PAX5 was shown to regulate several hundred target genes in pre-B, pro-B and mature B-cells, respectively [4, 5]. Thereby, PAX5 influences a variety of biological processes in B-cells, including transcriptional and cell-cycle control [5], B-cell differentiation [6] and metabolism [7] as well as cell adhesion and migration [8]. As both pro-B and mature B-cell target gene sets only minimally overlap, a complex network has to be in place to delicately balance PAX5 functionality between the different developmental stages of B-cell differentiation [5]. Accordingly, complete PAX5 loss leads to a block in B-cell differentiation at an early B-cell precursor stage, as seen in *Pax5* knockout mice (*Pax5*^−/−^) [9]. In addition, precursor B-cells of *Pax5*^−/−^ mice are characterized by hematopoietic stem-cell-like features, including self-renewal capacity and multipotency. Furthermore, conditional inactivation of *PAX5* in committed B-cells results in dedifferentiation with the potential of re-differentiation into other hematopoietic lineages [10, 11].

Hence, deregulated *PAX5* expression has major implications in physiologic hematopoiesis and poses a high risk for leukemia development, with somatic alterations being one of the most common features present in B-cell precursor acute lymphoblastic leukemia (BCP-ALL) [12]. Along these lines, two new BCP-ALL subtypes encompassing somatic *PAX5* aberrations - *PAX5*alt (7.4%) and *PAX5* p.P80R (2.2%) - have recently been discovered [13]. Here, *PAX5*alt is defined by a distinct gene expression profile with diverse *PAX5* alterations, including fusions, intragenic amplifications, or mutations (all except *PAX5* p.P80R) [13]. Somatic copy number alterations of *PAX5* in BCP-ALL, like deletions, are mostly mono-allelic and could therefore act as cooperating events in BCP-ALL [14]. In contrast, point mutations (e.g. *PAX5* p.P80R) and *PAX5*alt cases show predominantly bi-allelic sequence alterations, with loss of the *PAX5* WT allele, suggesting a driver role in BCP-ALL [14]. Particularly in the second scenario, this highlights PAX5 functionality as a barrier to malignant B-cell transformation [7]. Moreover, this suggests a stepwise process of *PAX5* inactivation during leukemia development, clearly recapitulated in familial BCP-ALL cases with heterozygous *PAX5* germline variants, including *PAX5* p.G183S [15, 16] and *PAX5* p.R38H [17, 18]. In this scenario, *PAX5* germline variants pose the first hit by conferring BCP-ALL susceptibility, whereas the second hit manifests through loss of heterozygosity (LOH) caused by chromosomal 9p aberrations (carrying the *PAX5* gene locus), which is somatically detectable in almost 100% of the respective tumors [19]. While the variable penetrance of BCP-ALL in these families can be explained by additional environmental influences, as previously shown by our group through infection exposures [20], the initial effect of *PAX5*-mediated leukemia predisposition is insufficiently understood. Additionally, while *PAX5* loss has a detrimental impact on B-cells, studies in heterozygous *Pax5* animals (*Pax5*^±^) have not yet shown a profound phenotype [9]. This is surprising because heterozygous germline alterations, including point mutations and deletions/frameshifts in *PAX5*, are associated with BCP-ALL in children with a high penetrance [14, 21–23], suggesting that even a mild reduction in *PAX5* activity can initiate clonal evolution to BCP-ALL. Although these children are initially known to respond, rather well, to conventional chemo- and immunotherapies, around 2/3^rds^ develop relapse, and most patients so far have undergone hematopoietic stem cell transplantation (HSCT) [19]. Families carrying *PAX5* germline alterations show phenotypically highly similar BCP-ALL with a median age of onset below 8 years of age (for p.G183S/R variants and other frameshifts/deletions) or latest up until young adulthood (for carriers of the p.R38H variant), indicating a genetically imposed limited time window of susceptibility (Supplementary Fig. 1A).

Hence, it is essential to reveal the precise impact of reduced *PAX5* transcriptional activity on B-cell precursors to distinguish healthy from pathological status at the earliest possible stage. This will allow [1] early prediction of BCP-ALL in families with a genetic predisposition and [2] understanding of the clonal evolution of BCP-ALL with acquired *PAX5* mutations. Both aspects will facilitate treatment at the earliest disease stages aiming for cure before clinical overt disease onset requiring targeted treatment regiments. This is key for early therapy or even prevention in both, PAX5-mediated familial BCP-ALL predisposition and somatically acquired PAX5-driven leukemias.

To address both of these questions, *Pax5*^±^ mice represent a suitable system, since they closely mimic the stepwise clonal evolution of BCP-ALL observed in families with *PAX5* p.G183S/R initiated BCP-ALL [19–23]. Thus, using this model, we present a comprehensive elucidation of the longitudinal developmental effect of gradual *PAX5* loss in BCP-ALL development at single-cell level, by integrating multicolor flow cytometry with bulk and single-cell genomic technologies. Subsequently, this study depicts PAX5-dependent checkpoints in single pre-leukemic cells interacting with MYC and identifies new avenues for BCP-ALL prevention.

## Results

### Single-cell RNA-Sequencing confirms CD25 as a suitable cell-surface marker to discriminate precursor B-cell subsets

Germline *PAX5* variants have been shown to predispose to BCP-ALL development with a high penetrance [19]. However, although *PAX5* is known to display bi-allelic expression at all stages of B-cell development [24], *Pax5* haploinsufficiency is believed to be phenotypically unremarkable without a biological effect on precursor and mature B-cells [9].

**Fig. 1:**
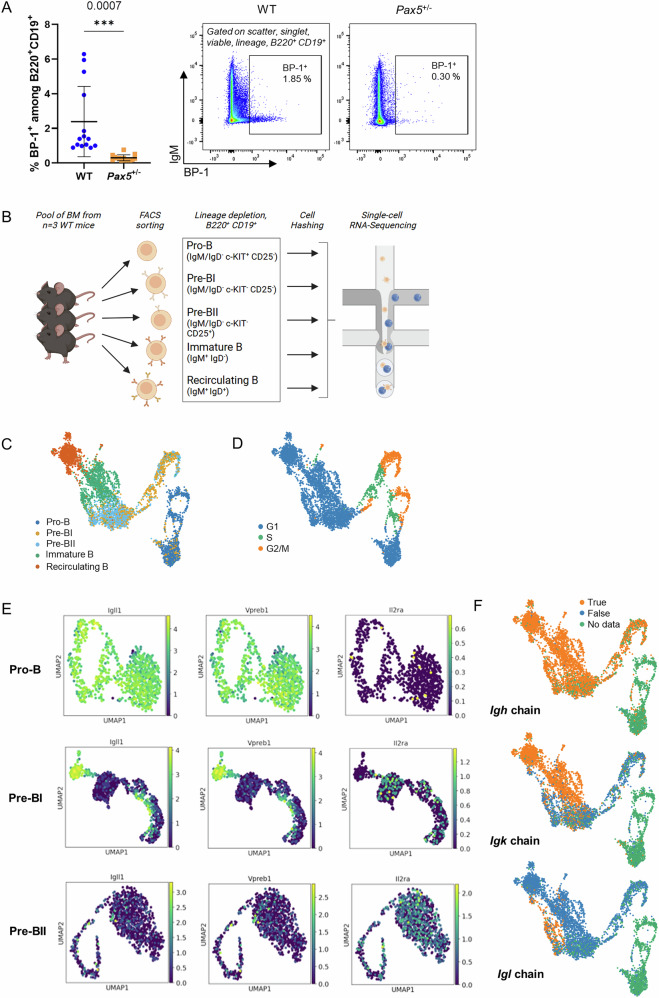
The Basel classification accurately resolves transcriptionally distinct B-cell precursor subsets in the murine bone marrow. **A** Percentage of BP-1^+^ bone marrow B-cells (B220^+^ CD19^+^) from *Pax5*^±^ vs. wild-type (WT) mice. (Left) Displayed are individual values (with mean and SD) of 14 *Pax5*^±^ vs. 14 WT mice ages 11-38 weeks. (Right) Representative flow cytometry plots. An unpaired two-tailed Student’s t-test was used to calculate significance and the respective *p*-value is indicated. *** *p* ≤ 0.001. **B** Schematic of the employed sorting and single-cell RNA-Sequencing strategy. **C** Single-cell clusters of different WT precursor B-cell subsets analyzed with the workflow depicted in B). The transcriptome similarity is visualized based on UMAP. Pool of *n* = 3 WT mice, 17 weeks of age. **D** Predicted cell-cycle state in clusters from C). **E** Precursor B-cells of WT mice display specific gene expression patterns depending on their differentiation state. The scaled gene expression level for classical marker genes (*Igll1*, *Vpreb1* and *Il2ra*) is shown in color on the UMAP. **F** Expressed immunoglobulin chain status of WT precursor B-cell clusters displayed as in C). Igh immunoglobulin heavy chain, Igk immunoglobulin kappa light chain, Igl immunoglobulin lambda light chain. True chain expression, False no chain expression, No data no chain detected.

To shed light on this discrepancy, we aimed to identify subtle but biologically relevant consequences of germline *Pax5* heterozygosity on the B-cell development in the bone marrow (BM), utilizing *Pax5*^±^ mice. Thus, using multicolor flow cytometry analysis, we first investigated distinct B-cell lineage subsets in the BM of *Pax5*^±^ vs. WT mice. Interestingly, the most commonly used Hardy B-cell classification [25] could not be applied as *Pax5*^±^ B-cell precursors showed significantly reduced levels of the cell surface marker BP-1 (*p* = 0.0007, Student’s *t*-test) (Fig. 1A). While the respective *Enpep* gene is a known target of PAX5, its deregulation on the cell surface has, so far, only been reported in response to complete *PAX5* loss. To circumvent B-cell subset discrimination via BP-1, the likewise popular Basel staining [26], separating precursor B-cells based on CD25 expression, was alternatively implemented (Supplementary Fig. 1B). To first validate whether this staining approach is feasible to segregate between different precursor B-cell developmental stages, both on a phenotypic and a transcriptional level, we performed single-cell RNA-Sequencing (scRNA-Seq) of Basel staining based sort-purified B-cell precursor subsets from the BM of WT mice (pool of 3 mice) as follows: pro-B (B220^+^ CD19^+^ c-KIT^+^ CD25^−^ IgM^−^ IgD^−^), pre-BI (B220^+^ CD19^+^ c-KIT^−^ CD25^−^ IgM^−^ IgD^−^), pre-BII (B220^+^ CD19^+^ c-KIT^−^ CD25^+^ IgM^−^ IgD^−^), immature B (B220^+^ CD19^+^ IgM^+^ IgD^−^) and recirculating B (B220^+^ CD19^+^ IgM^+^ IgD^+^) (Fig. 1B). The transcriptional clustering revealed a clear separation between most precursor B-cell stages (Fig. 1C). The cells designated as pre-BI showed only a minor proportion of cells assigned into clusters matching their preceding pro-B and succeeding pre-BII differentiation stages (Fig. 1C). Together with the observation that the majority of pre-BI cells are actively cycling, these data highlight the pre-BI cell stage as proliferating precursors on their transition from pro-B to pre-BII (Fig. 1D). This was further validated by the distinct expression of surrogate-light chain markers *Igll1* and *Vpreb1* in pro-B cells and in a small fraction of pre-BI cells, which were mutually exclusive to *Cd25* (*Il2ra*) expressing cells. In contrast, pre-BII clusters were almost entirely lacking *Igll1* and *Vpreb1* expression, and mostly expressed *Cd25* (Fig. 1E, Supplementary Fig. 1C). As independent confirmation, we incorporated V(D)J-receptor analysis, and this data additionally validated a clear separation of the differentiation stages based on rearrangement status. While immunoglobulin heavy chain (*Igh*) expression was first detected in pre-BI cells, immunoglobulin light chain expression (*Igk* and *Igl*) started from the pre-BII stage onward (Fig. 1F). Together, these data confirm that the employed phenotypic Basel classification based on CD25 expression is suitable to discriminate between transcriptionally different B-cell precursor subsets in the murine BM.

### Loss of one copy of *Pax5* leads to an aberrant pre-BII population

**Fig. 2:**
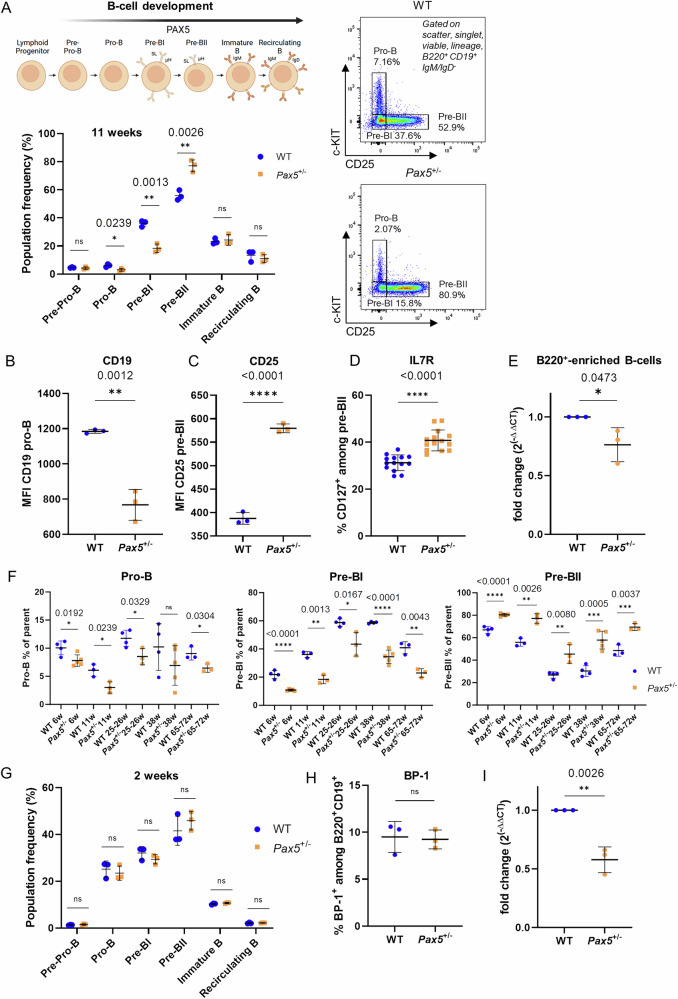
Aberrant precursor B-cell development in *Pax5*^+/−^ mice emerges postnatally and persists with age. **A** Upper: Schematic of B-cell differentiation in the bone marrow. SL surrogate light chain, µH heavy chain. Lower: Population frequencies among the different differentiation stages (individual values with mean and SD). Compared are wild-type (WT) mice (*n* = 3, 11 weeks) and *Pax5*^±^ littermates (*n* = 3, 11 weeks). Representative flow cytometry plots displaying the pre-BII population (B220^+^ CD19^+^ IgM^−^ IgD^−^ c-KIT^−^ CD25^+^) for WT and *Pax5*^±^ mice are displayed on the right. **B** Mean fluorescence intensity (MFI) of CD19 in pro-B cells of *Pax5*^±^ mice compared to WT littermates. **C** Analogous to B) for CD25 MFI in pre-BII cells. **D** Percentage of IL7-Receptor (IL7-R, CD127) positive cells among pre-BII cells of *Pax5*^±^ mice compared to their WT littermates. **E** qRT-PCR analysis showing *Pax5* gene expression in B220-enriched bone marrow cells of 14 weeks old *Pax5*^±^ mice compared to WT animals (*n* = 3). **F** Frequencies of pro-B, pre-BI and pre-BII cells within the (B220^+^ CD19^+^ IgM^−^ IgD^−^) parental population in WT vs. *Pax5*^±^ mice across different age cohorts (6 weeks, WT *n* = 4, *Pax5*^±^ *n* = 5; 11 weeks, WT *n* = 3, *Pax5*^±^ *n* = 3; 25–26 weeks, WT *n* = 4, *Pax5*^±^ *n* = 3; 38 weeks, WT *n* = 4, *Pax5*^±^ *n* = 5; 65–72 weeks, WT *n* = 3, *Pax5*^±^ *n* = 3). w weeks. **G** Population frequencies among the different differentiation stages. Compared are WT mice (*n* = 3, 2 weeks) and *Pax5*^±^ littermates (*n* = 3, 2 weeks). **H** Percentage of BP-1^+^ bone marrow B-cells (B220^+^ CD19^+^) from 2 weeks old *Pax5*^±^ vs. WT mice (*n* = 3 per group). **I** qRT-PCR analysis showing *Pax5* gene expression in B220-enriched bone marrow cells of 2 weeks old *Pax5*^±^ compared to WT animals (*n* = 3 per group). Displayed are individual values with mean and SD. An unpaired two-tailed Student’s t-test was performed for the statistical analysis. Respective *p*-values are indicated. ns not significant, *p ≤ 0.05, **p ≤ 0.01, ***p ≤ 0.001, ****p ≤ 0.0001.

Next, we used the Basel staining to compare the distribution between developing B-cells in the BM of *Pax5*^±^ and WT mice. Utilizing this approach, we observed an altered composition of multiple B-cell developmental stages. Specifically, while the percentages of the pro-B and pre-BI cell subsets were significantly reduced (*p* = 0.0239 and *p* = 0.0013 respectively, Student’s t-test), the pre-BII compartment was significantly enriched (*p* = 0.0026, Student’s t-test) in 11 week old *Pax5*^±^ mice compared to their WT littermate controls (Fig. 2A). Interestingly, no difference in population frequency was noted for both B-cell receptor (BCR) expressing subsets (immature B and recirculating B), suggesting that the majority of deregulations mediated through *Pax5* haploinsufficiency take effect at the precursor (pro-B, pre-BI and pre-BII) B-cell stage.

Along these lines the reduced pro-B cell population in *Pax5*^±^ mice displayed lower mean fluorescence intensity (MFI) of CD19 (*p* < 0.0012, Student’s *t*-test), a well-known target gene of PAX5 (Fig. 2B, Supplementary Fig. 2A). Furthermore, the aberrant pre-BII population identified in *Pax5*^±^ mice showed a strikingly higher CD25 MFI compared to their WT counterparts (*p* < 0.0001, Student’s t-test) (Fig. 2C, Supplementary Fig. 2B). Again, with CD25 being a known repressed PAX5 target gene [5], this signature can be directly attributed to *Pax5* haploinsufficiency. However, to exclude the possibility of mislabeled pre-BI cells that were artificially recorded under pre-BII due to de-repression of the CD25 surface marker in *Pax5*^±^ mice, we stained CD25 positive and negative B-cell subsets (CD19^+^B220^+^) for the surrogate light chain markers VPREB (CD179a) and λ5 (CD179b). While both cell surface markers were detected at equal levels in the CD25^−^ populations of *Pax5*^±^ and WT mice, they were strongly downregulated to comparable levels in the CD25^+^ cells of both *Pax5*^±^ and WT mice, validating their respective progressed developmental stage and therefore their true pre-BII identity (Supplementary Fig. 2C). Additionally, the enriched pre-BII population observed in *Pax5*^±^ mice displayed higher IL7R (CD127) expression compared to pre-BII cells in the WT condition (*p* < 0.0001, Student’s t-test) (Fig. 2D).

Next, we assessed whether the observed deregulated B-cell differentiation in *Pax5*^±^ mice is related to differences in *Pax5* expression levels. Quantitative Real-time PCR (qRT-PCR) analysis of B220-enriched B-cells from the BM of *Pax5*^±^ mice showed a reduction of overall *Pax5* transcript levels to around 70% compared to WT expression (Fig. 2E). The same reduction was validated in individually sorted B-cell precursor subsets (pro-B, pre-BI, pre-BII and immature B) (Supplementary Fig. 2D).

To investigate whether the observed phenotype within the precursor B-cell subsets is stable over time, BM B-cell populations of different cohorts of aging mice were analyzed via flow cytometry as described above. These results validated a reduction of pro-B and pre-BI cell percentages with a concomitant increase of the pre-BII population frequency in *Pax5*^±^ mouse cohorts aged 6 weeks, 11 weeks, 25–26 weeks, 38 weeks and 65-72 weeks, respectively (Fig. 2F, Supplementary Fig. 2E). The same trend could also be reproduced when plotting the frequencies of precursor B-cell subsets among viable cells instead of their frequencies among the parental population (Supplementary Fig. 2F). No consistent differences were observed within immature B or recirculating B-cell percentages among the different age cohorts (Supplementary Fig. 2G). As expected *Pax5* heterozygosity did not affect the hematopoietic stem and progenitor (HS/PC) compartment (Supplementary Fig. 2H), further highlighting its exclusive effect on early B-cell precursors. Interestingly, the analysis of a 2-week-old cohort, in which initial BM B-cell hematopoiesis is not yet fully complete as recirculating B-cells are still sparse (Supplementary Fig. 2I), showed no differences in pro-B, pre-BI and pre-BII cell populations between WT and *Pax5*^±^ mice (Fig. 2G). Moreover, in contrast to older mice, BP-1 percentages showed similar levels in WT and *Pax5*^±^ mice at 2-weeks of age (Fig. 2H). qRT-PCR analysis confirmed that *Pax5* expression levels were maintained at reduced levels (still at around 70%, similar to analyzed older mice) in the 2-week-old *Pax5*^±^ cohort (Fig. 2I). Taken together, our data show that loss of one single copy of *Pax5* imposes an aberrant development of precursor B-cells, which occurs after BM hematopoiesis reaches a steady-state and remains persistent with ageing.

### *Pax5* haploinsufficiency in pre-BII cells causes downregulation of essential BCR components

**Fig. 3:**
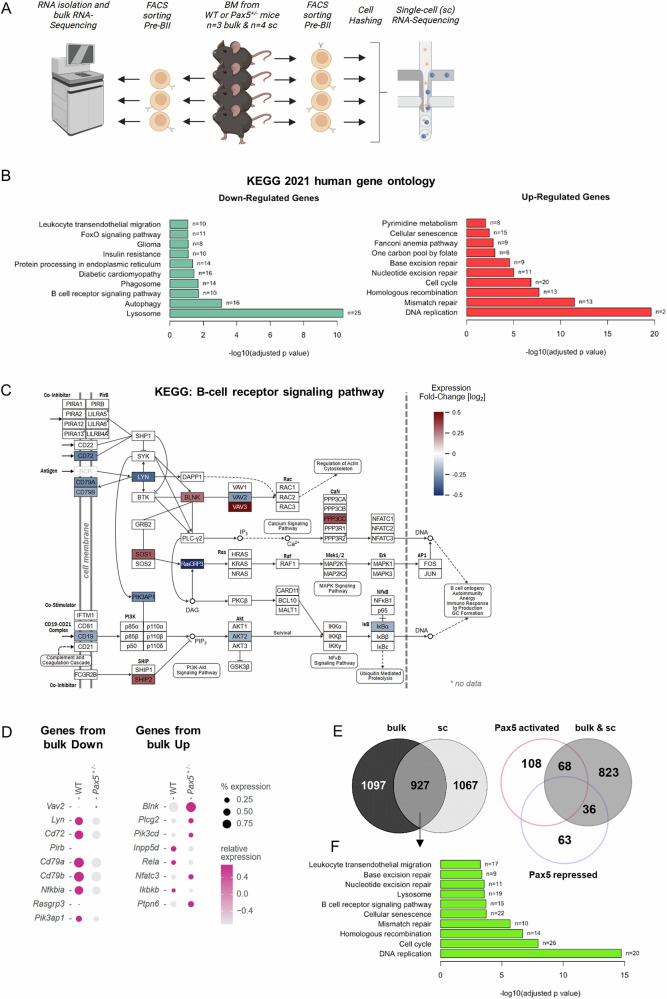
Integrated bulk and single-cell RNA-Seq reveal transcriptional dysregulation in *Pax5*^+/−^ pre-BII cells. **A** Analysis strategy for bulk and single-cell (sc) RNA-Sequencing of the pre-BII population. Bulk RNA-Sequencing: *n* = 3 wild-type (WT) and *n* = 3 *Pax5*^±^ littermates all aged 10 weeks. The same mice were used for scRNA-Sequencing together with one additional WT and *Pax5*^±^ mouse each, both aged 10 weeks. All 8 mice for scRNA-Sequencing were individually hash-tagged for scRNA-Seq. BM bone marrow. **B** GO-term analysis of significantly up- and downregulated genes in *Pax5*^±^ vs. WT mice identified by bulk RNA-sequencing (FDR = 2%) performed with the online tool Enrichr. The bar lengths represent the adjusted *p*-value [-log_10_]. **C** Regulation of gene expression of the B-cell receptor signaling pathway genes (KEGG database) in *Pax5*^±^ compared to WT pre-BII cells as identified in B). Graphical representation was taken from the KEGG database and manually adjusted to include respective mouse genes. **D** Visualization of scRNA-Seq data using the top 9 down- and the top 8 upregulated genes from bulk RNA-Seq analysis of *Pax5*^±^ and WT pre-BII cells as dot plots. Color tones correspond to average expression level and dot size represents percentage of cells expressing each gene. **E** (Left) Venn diagram showing the overlap of significantly deregulated genes in pre-BII cells from *Pax5*^±^ mice compared to WT controls between bulk (FDR = 10%) and scRNA-Seq data (*n* = 927). (Right) Venn diagram displaying the intersection between overlapping deregulated genes between bulk and scRNA-Seq to PAX5 targets in small pre-B cells (as identified by Fedl et al. [4]). **F** GO-term analysis of the overlapping genes (*n* = 927) from E) left Venn diagram.

While cell surface marker analyses are valuable to identify general developmental deregulations, they are not sufficient to fully recapitulate the intrinsic nature of the respective phenotype. Therefore, we next employed bulk and scRNA-Seq to further investigate transcriptional differences and potential heterogeneities within the pre-BII compartment of *Pax5*^±^ mice (Fig. 3A). Bulk RNA-Sequencing (pre-BII cells; 3 *Pax5*^±^ vs. 3 WT) confirmed clustering of cells from the same genotype and showed a total of 635 significantly upregulated and 621 significantly downregulated genes in pre-BII cells from *Pax5*^±^ compared to WT mice (false discovery rate (FDR) = 2%, see Methods Section and Supplementary Table 1, Supplementary Fig. 3A–C). As expected, *Pax5* expression was significantly reduced to around 70% in *Pax5*^±^ compared to WT pre-BII cells (Supplementary Fig. 3D), independently validating the previous results obtained from qRT-PCR analysis in sorted pre-BII cells (refer to Supplementary Fig. 2D). GO-Term analysis of regulated genes further revealed BCR signaling as one of the top downregulated pathways, while DNA replication and repair and cell-cycle signaling pathways were upregulated in *Pax5*^±^ pre-BII cells (Fig. 3B). Since pre-BII cells represent the final developmental stage before BCR assembly on the cell surface, proper expression of all BCR signaling components is crucial for a coordinated transition. In this regard our data revealed shortcomings of essential BCR signaling gene transcripts at the pre-BII stage in *Pax5*^±^ mice, including genes encoding *Cd79a/b*, *Cd19*, *Cd72* and *Lyn* (Fig. 3C and Supplementary Fig. 3E). Additionally, pre-BII cells of *Pax5*^±^ mice showed downregulation of a variety of BCR accessory proteins, e.g. immunoreceptor tyrosine-based inhibitory motifs (ITIMs), cholesterol metabolism related proteins as well as ribosomal and mitochondrial proteins (Supplementary Table 1).

The corresponding scRNA-Seq data (pre-BII cells; 4 *Pax5*^±^ vs. 4 WT) presented a high degree of concordance to the bulk RNA-Seq data. This was confirmed by the comparison of the top 10 down- and upregulated genes from bulk RNA-Seq in the scRNA-Seq data, displayed by transcript detection (shown in dot size) and expression level (shown in color) analyses across cells separated by genotype (Fig. 3D). Overall sc and bulk RNA-Seq yielded a total of 927 overlapping differentially regulated genes in pre-BII cells from *Pax5*^±^ mice compared to WT mice, out of which 68 and 36 were proven activated or repressed PAX5 target genes in small pre-B cells, respectively, when compared to recently published data [4] (Fig. 3E, Supplementary Table 2). Pathway analyses, which considered the intersection between both methods, again displayed enrichments in deregulated genes involved in DNA replication, cell-cycle, DNA repair and BCR signaling (Fig. 3F). In summary, loss of one copy of *Pax5* leads to deregulated BCR-assembly and expression together with aberrations in cell-cycle and DNA replication/repair mechanisms, posing the cells for malignant transformation upon additional genetic insults.

### Pre-BII cells of *Pax5*^±^ mice display defects in BCR light chain recombination

**Fig. 4:**
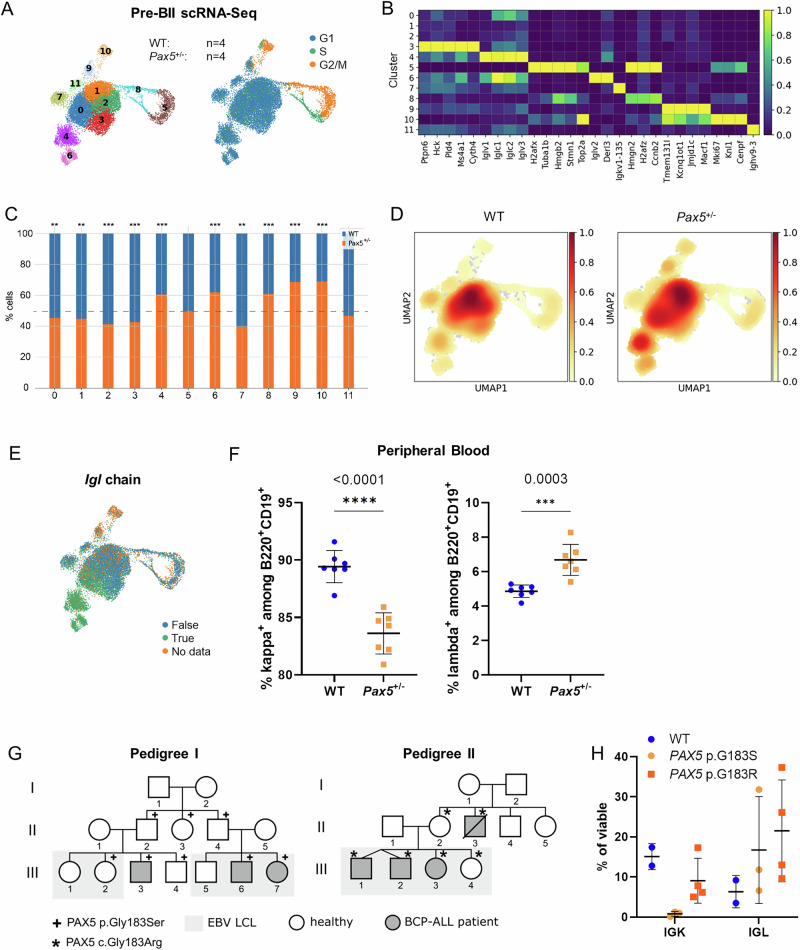
*Pax5* haploinsufficiency drives aberrant kappa/lambda light chain rearrangement in pre-BII cells. **A** Single-cell (sc) RNA-Sequencing clusters of sorted pre-BII cells from *n* = 4 wild-type (WT) and *n* = 4 *Pax5*^±^ mice ages 10-11 weeks (left) with annotated cell-cycle states (right). **B**: Top gene expression is shown for each cluster as a heatmap. **C** Cluster specific proportion of pre-BII cells from WT vs. *Pax5*^±^ mice. **D** Proportion heatmaps showing the cell distribution of pre-BII cells among the different clusters from *Pax5*^±^ and WT mice. The lower left clusters 4 and 6 (see Fig. 4A) correspond to lambda light chain recombination status in the V(D)J data (refer to Supplementary Fig. 4A). **E** V(D)J-recombination analysis showing annotation of immunoglobulin lambda light chain (*Igl*) expression. True chain expression, False no chain expression, No data no chain detected. **F** Flow cytometry analysis showing the percentage of kappa and lambda light chain rearranged B-cells among B220^+^ CD19^+^ cells in the peripheral blood of *Pax5*^±^ (*n* = 7) vs. WT (*n* = 7) mice ages 11–16 weeks (individual values with mean and SD). Significances (indicated) were calculated using an unpaired two-tailed Student’s t-test. ***p ≤ 0.001, ****p ≤ 0.0001. **G** Pedigrees of two families harboring *PAX5* germline variants (p.G183S and p.G183R, respectively). Epstein Bar Virus (EBV) transformed lymphoblastoid cell lines (LCL) were available from all individuals shaded in grey. BCP-ALL B-cell precursor acute lymphoblastic leukemia. **H** Flow cytometry analysis of IGK (immunoglobulin kappa light chain) vs. IGL surface expression levels on EBV LCLs depicted in **G**). *PAX5* p.G183S *n* = 3, *PAX5* p.G183R *n* = 4, *PAX5* WT *n* = 2.

To further investigate the biological influence of *Pax5* heterozygosity within the enriched pre-BII population, we analyzed cluster specific gene expression changes within the obtained scRNA-Seq data of sorted pre-BII cells from *Pax5*^±^ and WT controls (pre-BII cells; 4 *Pax5*^±^ vs. 4 WT) (Fig. 3A). Interestingly, although stringently refined by flow cytometry assisted cell sorting, scRNA-Seq revealed various sub-developmental stages (11 clusters) within the pre-BII compartment (Fig. 4A, B). Thereof, without proper *IgH* rearrangements clusters 9 and its cycling counterpart 10 represented a small subset of more pro-B-like cells (Supplementary Fig. 4A). The additional cycling clusters 5 and 8 with upregulated *Top2a* and *Ccnb2* expression on the other hand showed *IgH* transcripts, but only low expression levels of light chains, marking them as freshly transitioned pre-BI cells (Fig. 4B, Supplementary Fig. 4A). The remaining clusters were mainly separated based on their light chain signatures, with *Igk* positive cells in clusters 1, 2, 3, 7 and 11, and *Igl* positive populations in clusters 0, 4 and 6 (Fig. 4B).

Next, the clusters were mapped according to the individual mice and genotype groups. Subsequently, normalized percentages of cells from WT vs. *Pax5*^±^ mice per cluster showed a statistically significant difference between *Pax5*^±^ and WT pre-BII cells in all clusters except 5 and 11 (Chi squared test, Fig. 4C, D, Supplementary Table 3). Furthermore, *Pax5*^±^ pre-BII cells displayed a particular enrichment in the clusters 4 and 6 (Supplementary Table 3, Fig. 4D), which showed a high expression of *Igl* (Fig. 4E). Accordingly, flow cytometry analysis of an independent mouse cohort validated significantly reduced B-cell (B220^+^ CD19^+^) percentages with IGK, and significantly elevated B-cell percentages with IGL expression in the peripheral blood (PB) and BM of *Pax5*^±^ mice compared to their WT littermates (PB IGK: *p* < 0.0001 and IGL: *p* < 0.0003; BM IGK: *p* < 0.0251 and IGL *p* < 0.0246, Student’s t-test) (Fig. 4F and Supplementary Fig. 4B). However, the abnormal rearrangement ratio did not phenotypically manifest through changes in proliferation, as no specific differences in the cell-cycle between B-cell precursors from *Pax5*^±^ vs. WT mice could be observed (Supplementary Fig. 4C). Accordingly, *Myc* levels remained low/undetectable in *Pax5*^±^ cells (Supplementary Table 1).

To translate these findings to the human scenario, we additionally analyzed the percentage of kappa and lambda light chain rearranged B-cells in EBV-transformed lymphoblastoid cell lines (LCL) from two families harboring *PAX5* germline variants p.G183S and p.G183R [15, 19] (Fig. 4G, H). Although our data displays a trend towards IGL skewing in analogy to the murine data, only two children harboring germline *PAX5* WT in both alleles were available within the families, limiting the power of statistical analysis (Fig. 4H). Utilizing allele specific qRT-PCR and Western blotting, we could confirm that both the *PAX5* WT and the p.G183S variant alleles were expressed to similar levels and that the overall amount of PAX5 protein did not differ between WT or *PAX5* variant LCLs (Supplementary Fig. 4D, E). Taken together, these data suggest defects in proper kappa/lambda light chain rearrangement leading to aberrant light chain ratios upon *Pax5* germline deregulation.

### The deficit in BCR assembly delays the progression of the pre-BII to the immature B-cell stage

**Fig. 5:**
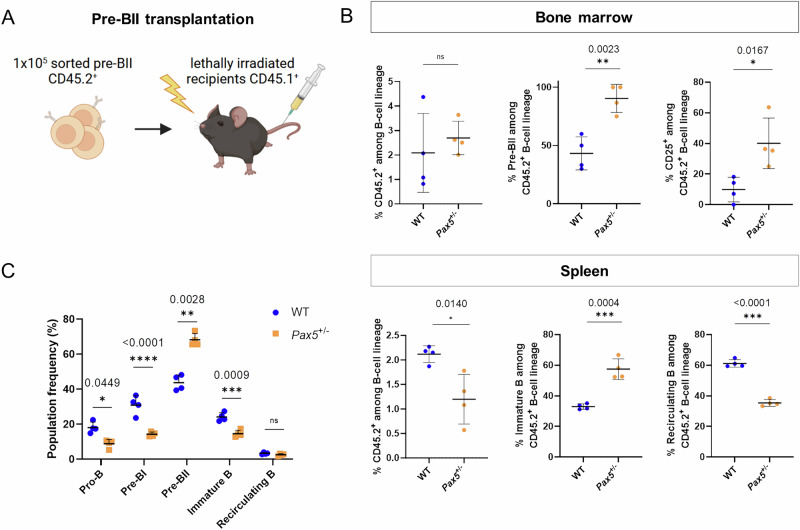
*Pax5* haploinsufficiency delays B-cell maturation and alters pre-BII cell homing in a transplantation model. **A** Schematic of transplantation strategy. **B** Flow cytometry analysis showing the percentage of engrafted donor CD45.2^+^ pre-BII cells in lethally irradiated recipients 72 h after transplantation in the bone marrow (upper) and the spleen (lower). Donor cells were a pool of *n* = 4, 11 weeks old either *Pax5*^±^ or wild-type (WT) mice, each transplanted into *n* = 5 CD45.1^+^ lethally irradiated recipients. No support whole bone marrow cells (WBMCs) was used for this experimental setup. “Among B-cell lineage” refers to gating on scatter, singlets, viable, lineage and B220^+^ CD19^+^. **C** Population frequencies of precursor B-cell subsets 72 h after in vitro cultivation of B220^+^ sorted cells from WBMCs of WT (*n* = 4) vs. *Pax5*^±^ (*n* = 4) mice, ages 9-10 weeks. Displayed are individual values with mean and SD. An unpaired two-tailed Student’s *t*-test was performed for the statistical analysis. Respective *p*-values are indicated. ns not significant, *p ≤ 0.05, **p ≤ 0.01, ***p ≤ 0.001, ****p ≤ 0.0001.

To question whether the transcriptional observations of deregulated BCR assembly at the pre-BII stage in *Pax5*^±^ mice result in a biological effect, we generated a transplantation model to study the differentiation potential of *Pax5* heterozygous pre-BII cells in vivo. Therefore, we sorted and transplanted 1×10^5^ pre-BII cells from *Pax5*^±^ or WT controls into lethally irradiated congenic recipients (Fig. 5A). 72 h after transfer, the BM and spleen of the recipient mice were analyzed, with CD45 staining being used to discriminate between donor (CD45.2^+^) and recipient (CD45.1^+^) cells. Our results show that significantly less pre-BII cells from *Pax5*^±^ mice matured and migrated to the spleen, but that they preferably homed to the BM as depicted by the percentage of pre-BII cells among CD45.2^+^ B-cells in the BM and their respective CD25 positivity (Fig. 5B). Accordingly, the progeny of donor CD45.2^+^ pre-BII cells from *Pax5*^±^ mice detected in the recipient’s spleen were mostly immature B, rather than recirculating B, suggesting delayed B-cell differentiation compared to PAX5 proficient (WT) pre-BII donor cells (Fig. 5B). In line with previous in vivo analyses, no differences in IgM^+^ fractions were observed in the BM between transplanted CD45.2^+^ pre-BII cells of *Pax5*^±^ and WT mice (Supplementary Fig. 5A). Along these lines, B220-enriched BM cells from *Pax5*^±^ mice cultured in vitro for 72 h maintained a similar pre-BII enrichment as observed in vivo, while they additionally displayed a significant reduction of immature B-cells (Fig. 5C) (*p* = 0.0009, Student’s t-test).

Subsequent assessment of transplanted pre-BII cells following longer tracking time, showed the absence of detectable cells in the BM 20 days after transplantation (data not shown), suggesting that the donor cells either died or were able to fully differentiate. Along these lines, transplantation of 1 × 10^5^ *Pax5*^±^ pre-BII cells (*n* = 5 receiving *Pax5*^±^ and *n* = 5 receiving WT cells) did not give rise to leukemia development in an aging mouse cohort, which was monitored up to a mouse age of 82 weeks (Supplementary Fig. 5B). Taken together, these data emphasize that loss of one *Pax5* copy still enables proper BCR assembly but delays B-cell maturation.

### Pre-leukemic cells of *Pax5*^±^ mice lose their B-cell identity and display high *Myc* levels

**Fig. 6:**
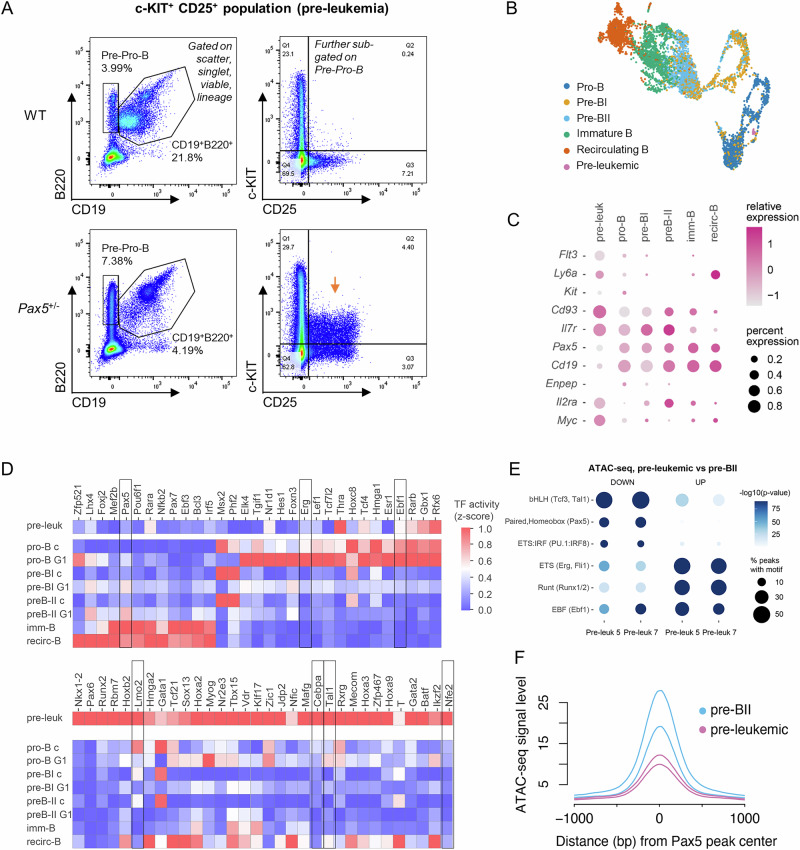
Pre-leukemic c-KIT^+^ CD25^+^ cells in *Pax5*^+/−^ mice exhibit *Myc* activation and multi-lineage transcription factor rewiring. **A** Representative flow cytometry plots showing a low percentage of a deregulated population (c-KIT^+^ CD25^+^) in the bone marrow of a 38 weeks old *Pax5*^±^ mouse (highlighted with arrow; termed pre-leukemia). This was accompanied with an enrichment of pre-pro B cells (B220^+^ CD19^−^). WT wild-type. **B** Single-cell RNA-Sequencing (scRNA-Seq) analysis including WT precursor B-cell subsets from Fig. 1C and the pre-leukemic population identified in A). Pre-leukemic cells cluster closest to WT pro-B cells. **C** Gene expression profile of selected genes in pre-leukemic cells (pre-leuk) compared to WT B-cell differentiation stages extracted from the scRNA-Seq analysis as a dot plot heatmap. Cluster labels are displayed in B). **D** Transcription factor (TF) activities in pre-leukemic cells compared to WT B-cell subsets based on the SCENIC analysis. Important TF regulons that are involved in hematopoietic cell differentiation identity are highlighted. See also Supplementary Fig. 6 showing the predicted activity of PAX5 and EBF1 regulons within our recorded scRNA-Seq data of different WT B-cell subsets on the UMAP. Imm-B Immature B-cells, Recirc-B Recirculating B-cells, c cycling. **E** Three most significantly enriched motif classes are shown from differentially accessible peaks comparing *Pax5*^±^ pre-leukemic (pre-leukemia 5 and 7) and WT pre-BII cells (up/down: higher/lower accessibility in pre-leukemia, respectively, refer to Supplementary Table S4). The dot size corresponds to the fraction of peaks carrying the motif. Color denotes -10log(*p*-value). **F** Histogram of ATAC-seq signal level at PAX5 binding sites comparing *Pax5*^±^ pre-leukemic (*n* = 2, pre-leukemia 5 and 7) and WT pre-BII (*n* = 2) chromatin access. Peaks centered with PAX5 motif are shown (*n* = 963 peaks, ± 1 kb from center, re-analysis of CUT & RUN data from pro-B and pre-B cells [4]).

Our data show that precursor B-cells of *Pax5*^±^ mice, albeit deregulated, still partially retain their differentiation capacity. Thus, during leukemia evolution additional events are necessary to arrest these precursors in a permanent susceptible de-differentiated state (termed pre-leukemia). To identify cells in a pre-leukemic state, we analyzed the BM of healthy *Pax5*^±^ mice of different ages housed in specific pathogen free (SPF) conditions for aberrant cell surface marker expressions. Interestingly, a fraction of aging *Pax5*^±^ mice (38 to 70 weeks) displayed an unusually high percentage of pre-pro-B cells (B220^+^ CD19^−^) (Fig. 6A). Further in-depth analysis of this population revealed a small, but clearly distinguishable c-KIT^+^ CD25^+^ cell population, which was absent in all other analyzed healthy WT and *Pax5*^±^ animals of the same age (Fig. 6A). scRNA-Seq analysis of this c-KIT^+^ CD25^+^ cell population showed a clustering with the pro-B cell signature (Fig. 6B). However, a more detailed comparison to WT pro-B cells based on marker genes typically expressed in BM B-lineage progenitors revealed an upregulation of very early B-cell markers, including *Flt3*, *Ly6a*, *Cd93* and *Il7r* (Fig. 6C). Furthermore, the percentage of *Pax5* expressing cells within the c-KIT^+^ CD25^+^ cell population was lower, which was confirmed by downregulation of *Cd19*, *Enpep* and upregulation of *Il2ra* (Fig. 6C). Additionally, the cells displayed a strikingly higher *Myc* level than any other B-cell developmental stage in the WT BM, including *Pax5*^±^ pre-BII cells (Fig. 6C, Supplementary Fig. 6A). Based on these findings, we termed the c-KIT^+^ CD25^+^ cell population a pre-leukemic population. Downregulation of *Pax5* and upregulation of *Myc* was validated via qRT-PCR analysis in 2 additional independent *Pax5*^±^ pre-leukemic mice (pre-leukemia 5 and 7) displaying the c-KIT^+^ CD25^+^ cell population (Supplementary Fig. 6B). Furthermore, we collected samples from these same pre-leukemic mice and WT pre-BII cells for the analysis of chromatin accessibility at the respective gene loci using ATAC-seq. In both c-KIT^+^ CD25^+^ cell populations, we could distinguish high chromatin access at the distal Myc enhancer that is bound by EBF1 and PAX5 in WT pro-B cells but has no binding and low chromatin access in WT pre-B cells (as shown through re-analysis of publish CUT & RUN data from Fedl et al.) (Supplementary Fig. 6C).

To discover additional TFs that may shape the pre-leukemic chromatin landscape in this population, we inferred TF activity from gene expression data using TF activity scoring based on Scenic analysis [27] (Supplementary Fig. 6D) comparing the identified pre-leukemic c-KIT^+^ CD25^+^ cell population in the *Pax5*^±^ mouse from Fig. 6A to murine WT B-cell precursors (Fig. 6D), and as an independent confirmation used the chromatin accessibility profiles, which were acquired in the two additional independent pre-leukemic samples (pre-leukemia 5 and 7). Confirming that loss of *Pax5* activity contributes to the pre-leukemic state, these cells had diminished *Pax5* TF activity score (Fig. 6D) and showed enrichment of the respective motif in ATAC-seq peaks with lower chromatin access compared to WT pre-BII cells (Fig. 6E, Supplementary Table 4). In further confirmation, the chromatin accessibility signal was strongly decreased at experimentally defined direct binding sites for PAX5 in pre-leukemic cells compared to WT pre-BII cells (Fig. 6F). In addition, peaks with lower chromatin access included bHLH and ETS:IRF motifs. In contrast, the higher TF activity of *Ebf1*, *Erg* and *Runx1/2*, could be confirmed as enrichment of their respective motifs (EBF, ETS and Runt, respectively) in peaks with higher chromatin access **(**Fig. 6E, Supplementary Table 4), verifying a de-differentiated signature with expression of pro-B TFs, including EBF1, a known activator of *Myc* expression [28]. In addition, the c-KIT^+^ CD25^+^ cell population showed high TF activity scores for master regulator TFs corresponding to a multipotent stage (Fig. 6D, bottom panel). Matching the scRNA-seq results, the chromatin landscape was modulated at several lineage specification TF and early B cell marker gene loci (e.g. TSS regions of *Cebpa*, *Tal1*, *Nfe2* as well as TSS and enhancers of *Lmo2* Supplementary Fig. 6E, F, Supplementary Table 4). Taken together, these data show that further reduction of *Pax5* transcriptional activity leads to the inability of precursor B-cells to progress to mature B-cells, multi-lineage TF expression, as well as a dominant *Myc* signature.

### BCP-ALLs from *Pax5*^±^ mice are heterogeneous in their expression profile but maintain high *Myc* expression

**Fig. 7:**
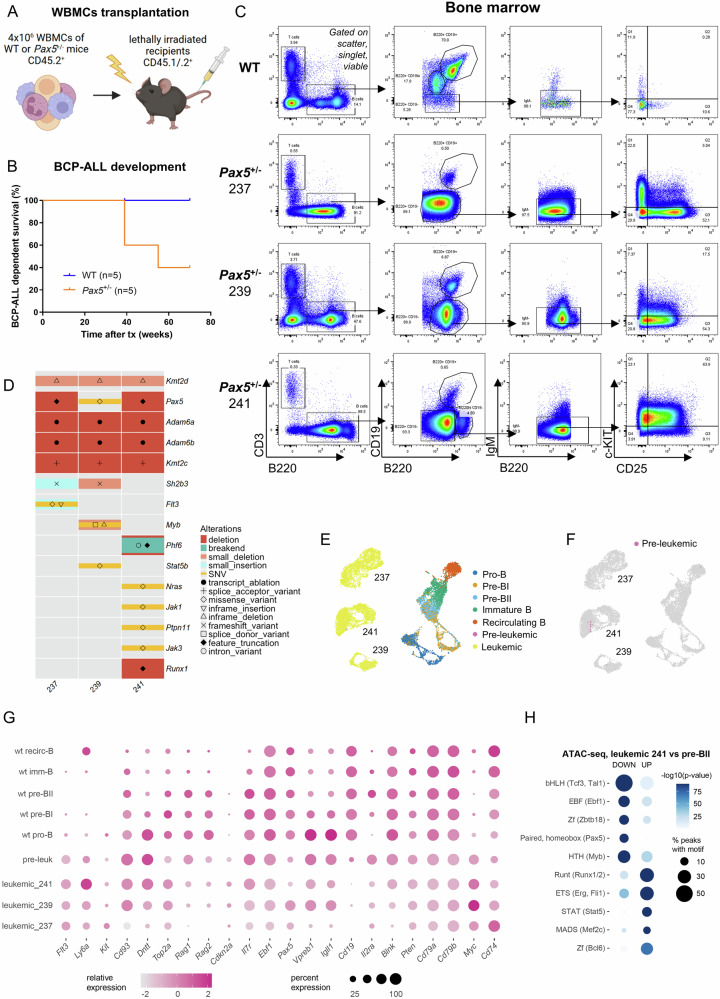
BCP-ALLs derived from *Pax5*^+/−^ mice display JAK/STAT mutations, *Pax5* loss, and chromatin remodeling linked to *Myc* activation. **A** Schematic of transplantation strategy. WBMCs whole bone marrow cells, WT wild-type. **B** B-cell precursor acute lymphoblastic leukemia (BCP-ALL) dependent survival curve of *n* = 5 CD45.1/.2 lethally irradiated recipients per genotype, transplanted each with 4 × 10^6^ WBMCs from either WT or *Pax5*^±^ mice (pool of 4 donor mice/donor genotype, 11 weeks of age). tx = transplantation. **C** Flow cytometry bone marrow characterization of the leukemias developed in B). **D** Selected somatic mutations and structural variants identified in the *Pax5*^±^ BCP-ALLs through whole genome sequencing (WGS) shown as an oncoprint heatmap with variant types annotated by color and shape. **E** Single-cell (sc) RNA-Sequencing of *Pax5*^±^ leukemias (237, 239 and 241) displayed together with sc expression profiles from WT B-cell subsets and pre-leukemic cells (Fig. 6B) on the UMAP. **F** Pre-leukemic cells cluster closest to leukemia 241. **G** Dot plot heatmap showing gene expression for selected genes across all analyzed B-cell subsets during BCP-ALL evolution. Imm-B Immature B-cells, recirc-B Recirculating B-cells, pre-leuk pre-leukemic. **H** Five most significantly enriched motif classes are shown (as in Fig. 6E) from differentially accessible peaks comparing *Pax5*^±^ leukemic (BCP-ALL 241) and WT pre-BII cells.

Lastly, we set out to explore the gene expression signature of full-blown BCP-ALLs arising from *Pax5* heterozygosity. It was previously shown that around 25% of *Pax5*^±^ mice develop BCP-ALL following exposure to infection, which was mediated through non-specific pathogen free (conventional) animal housing [20]. Based on these data we postulated that various external stressors, which usher precursor B-cells to undergo maturation, can trigger BCP-ALL on the basis of diminished *Pax5* expression levels. Hence, rather than the undefined stress of infection exposure, we utilized whole bone marrow cells (WBMCs) transplantation into irradiated recipients as an alternative experimental setup. As hypothesized, the imposed stress of repopulating the entire hematopoietic system after irradiation increased the incidence rate of BCP-ALL to 60%, with 3 out of 5 recipients of *Pax5*^±^ WBMCs developing the disease within 9 months after transplantation (Fig. 7A, B). The leukemias displayed a precursor B-cell surface phenotype of B220^+^ CD19^−^ IgM^−^ together with variable c-KIT and CD25 expression (Fig. 7C).

Whole genome sequencing (WGS) revealed several structural variants (SVs) in each of the BCP-ALLs (Fig. 7D and Supplementary Tables 5 and 6). We reasoned that these could represent early mutation events mediated by prolonged RAG-activity at the pre-BII stage. To investigate this further, we performed RAG consensus motif annotation of breakpoint sequences. As expected, this revealed multiple unique on-target rearrangements within the genomic region encoding light chains in each BCP-ALL (Supplementary Table 5). Moreover, recurrent off-target rearrangements harboring the recombination signal sequences (RSS) motif were found in *Kmt2c*, disrupting the coding sequence (Supplementary Table 7). In addition, cells from leukemia 241 carried deletions in *Runx1* and *Phf6* (Fig. 7D and Supplementary Table 5). Likewise, as expected through the absence of CD19, all three BCP-ALLs displayed genomic aberrations in *Pax5* (Fig. 7D and Supplementary Table 5).

Larger deletions included a recurrent loss of *Adam6a* and *Adam6b* which are also found in human ALL (Supplementary Table 6) [29]. Among single nucleotide variants (SNV) and indel type mutations, we found recurrent somatic mutations affecting the JAK-STAT and NRAS signaling pathway in all three BCP-ALLs (Supplementary Table 5). Interestingly, leukemia 241 showed concomitant JAK3 (p.R653H) and NRAS (p.G13S) activation (Fig. 7D). Furthermore, all BCP-ALLs carried an indel in *Kmt2d*, known to cooperate with Kmt2c in driving leukemogenesis and additional indel and missense variants were also found in *Kmt2a* and *Kmt2b* (Supplementary Table 6) [30].

scRNA-Seq analysis showed a clear clustering of all BCP-ALLs apart from each other and apart from healthy WT precursor B-cells in the BM (Fig. 7E, F, Supplementary Fig. 7A). Surprisingly, although all BCP-ALLs originated from the same pool of transplanted *Pax5*^±^ WBMCs, their cell-cycle state composition showed striking differences. While two of the three leukemias (239 and 241) were mostly cycling in G2/M and S, most cells from 237 displayed a G1 arrest (Supplementary Fig. 7A, B). Single-cell gene expression analyses confirmed this heterogeneity (Fig. 7G, Supplementary Fig. 7C, D). Here, leukemia 237 showed particularly high levels of CD74. Leukemia 239 on the other hand displayed some remaining *Pax5* reads, although these might not necessarily correspond to a functional full-length transcript. Finally, leukemia 241 stood out through high *Ly6a* (*Sca1*) and *Dntt* expression and very little remaining *Pax5* transcript levels. In line with *Pax5* reduction, all BCP-ALLs showed *Cd25* (*Il2ra*) expression, while *Cd19* transcripts were almost completely absent. Compared to pre-leukemic cells, the full-blown leukemias harbored very low *Rag1* levels (see pre-leukemic stage Fig. 6 and Fig. 7G).

To discover changes upon leukemic transformation at chromatin level, we performed ATAC-seq on the BCP-ALL 241 cells (Fig. 7H). Matching the activated JAK/STAT-signaling mutation profile, we found enrichment of STAT-motifs in upregulated chromatin regions compared to WT pre-BII cells, together with RUNX and ETS motifs. The decreased access to bHLH (Tcf) and PAX motifs was preserved from pre-leukemia. However, also the EBF motif was enriched in the downregulated peaks (Fig. 7H).

Similar to the pre-leukemic stage, all BCP-ALLs displayed pronounced *Myc* expression compared to healthy WT BM B-cell differentiation stages (Fig. 7G), which according to our ATAC-seq profiles is driven by several highly active enhancer regions in BCP-ALL 241 (Supplementary Fig. 7E). Along these lines, the analyzed pre-leukemic cells clustered together with BCP-ALL 241 (Fig. 7F). Thus, *Myc* transcript expression, which was close to absent in healthy B-cell precursors, marked the transition to malignancy and maintained higher levels even following BCP-ALL transformation.

## Discussion

Knowledge on genetic predisposition in patients with leukemia is increasing rapidly but variable penetrance of disease hampers currently strategies for early intervention. Thus, early recognition of evolving single pre-malignant and malignant clones is fundamental for future intervention programs to prevent disease or treat at the earliest disease stages. *PAX5* has been known as a hallmark for BCP-ALL development for more than a decade [12]. While somatic *PAX5* alterations are found in every 3^rd^ B-cell leukemia [13], families harboring *PAX5* germline variants represent a rare but recurrent scenario, serving as a model for genetic predisposition and familial BCP-ALL [19]. In this context, our results elucidate the effect of PAX5 reduction in healthy B-cells and shed light on its role in the progression to malignant transformation.

Physiologically, during B-cell development, precursor B-cells start to rearrange their heavy chains at the pro-B cell stage [31]. Subsequently, in pre-B cells, the heavy chain rearrangement is completed and the respective transcript is assembled on the cell surface together with the surrogate light chains VPREB and λ5 to form a transient complex termed pre-B cell receptor (pre-BCR) [31–33]. After initial receptor testing, positive feedback loops trigger cell proliferation and promote allelic exclusion at the Ig-heavy chain locus [34]. Finally, late pre-B cells undergo light chain gene rearrangement to allow the expression of a fully matured BCR (IgM) at the immature B-cell stage [31]. Here, our scRNA-Seq data validated this sequential differentiation in the CD25-based sorted cell populations of WT mice.

In the current work, we show that *PAX5* haploinsufficiency by itself alters the distribution of B-cell precursors in a healthy BM environment. While most B-cell differentiation processes are tightly controlled by PAX5, only complete PAX5 loss is known to cause a significant effect on B-cell development, manifested by a differentiation block at the pro-B cell stage as first described in *Pax5*^−/−^ mice [9]. *Pax5* heterozygous mice on the other hand, are known to live healthy under SPF-conditions and to display a fully functioning B-cell lineage [9]. Nevertheless, when crossed to additional leukemia predisposing backgrounds (e.g. *Ebf1* or *Ikzf1* [35], *Stat5b* [36] or *BCR-ABL*^*p190*^ [37]) or challenged through external stressors (e.g. infection exposure [20] or antibiotics [38]) *Pax5*^±^ mice develop an aggressive BCP-ALL that closely resembles the human disease.

By utilizing our validated B-cell staining strategy, we demonstrate multiple effects on cell surface markers and gene expression, which is mediated through the loss of one copy of *Pax5*. The most striking differences on the cell surface included the downregulation of BP-1 (*Enpep*) and the upregulation of CD25 (*Il2ra*), which are both positively and negatively regulated by PAX5, respectively [5]. Overall, our analysis showed a reduction of the percentage of pro-B and pre-BI cells and an enrichment of pre-BII cells in *Pax5*^±^ mice vs. WT littermates. Here, pre-BII cells displayed particularly high expression levels of CD25. As CD25 upregulation is commonly used to monitor PAX5 loss [39], the increased CD25 expression in the pre-BII population of *Pax5*^±^ mice suggests a strong PAX5 dependency at this stage. Pre-BII cells are on the verge of BCR expression, which in turn relieves them from their IL7R dependency for survival [40]. Consequently, the enriched pre-BII population in *Pax5*^±^ mice showed higher IL7R expression compared to pre-BII cells from WT mice, further suggesting an intrinsic developmental adaption and proposing a delay in BCR expression on *Pax5*^±^ pre-BII cells. This hypothesis was validated by bulk and scRNA-Seq expression profiles with pre-BII cells of *Pax5*^±^ mice exhibiting downregulation of essential BCR components. With CD25 being described to play a critical role in BCR-feedback regulation via the recruitment of SHIP1 and SHP1 phosphatases, the observed deregulated BCR signaling could potentially be linked to the increased CD25 expression identified on *Pax5*^±^ pre-BII cells [41]. Together, these data suggest that *Pax5* heterozygosity delays the transition from the pre-BII to the immature B-cell stage, a finding further confirmed by in vivo transplantation assays showing preferential homing of *Pax5*^±^ pre-BII cells to the BM rather than the spleen, as well as by in vitro studies depicting delayed differentiation of cultured *Pax5*^±^ pro-B cells into immature B-cells.

Interestingly, the pre-BII enrichment observed in mice aged 6 to 72 weeks was not seen in young mice at 2 weeks of age, suggesting that the effects of *Pax5* heterozygosity only manifest after steady-state hematopoiesis has been reached. This occurred independently of *Pax5* expression levels, which remained consistently at approximately 70% in *Pax5*^±^ mice compared to WT, across both young and old mice and across various B-cell precursor subsets. Since, Pax5 is known to be bi-allelically expressed throughout BM B-cell development [24], our data indicate that the remaining WT allele in *Pax5*^±^ mice partially compensates for the loss of the second allele. This compensation was only observed in *Pax5*^±^ mice but not in *PAX5* p.G183S Mutant LCLs since the mutant allele could generate a translated PAX5 protein with reduced transcriptional activity and the overall PAX5 protein level in those cells was comparable to WT. The observed compensation in *Pax5*^±^ mice may result from transcriptional feedback mechanisms or post-transcriptional regulation, such as increased mRNA stability or translational efficiency. However, further studies are needed to elucidate the precise underlying mechanisms.

Additionally, our scRNA-Seq analysis of the *Pax5* haploinsufficient pre-BII population revealed a vast heterogeneity present within already pre-defined subsets of B-cell precursors and further emphasize the delicate balance that needs to be maintained by B-cell TFs at this stage. Along these lines, we revealed a skewing of the kappa/lambda light chain ratio towards *Igl* BCR rearrangements in *Pax5*^±^ pre-BII cells, which could be independently validated both in the BM and the PB of *Pax5*^±^ mice. A similar trend towards IGL preference was observed in EBV-transformed PB of families carrying *PAX5* germline variants. Here, we acknowledge that fresh blood from the respective families would be a more suitable system, since EBV transformation by itself might not have uniformly selected both kappa and lambda rearranged B-cell clones [42]. However, due to the rarity of families carrying *PAX5* germline variants this validation has to be addressed in future studies. Nevertheless, it was previously shown that PAX5 is important for V(D)J-recombination events since the frequency of *Igh* rearrangements from DJ to V(D)J was reduced to 1/50 in PAX5-deficient precursor B-cells [43]. Additionally, PAX5 is required for the recombination of distal V_H_ gene segments [44]. The here identified recombination defect in *Pax5*^±^ pre-BII cells is further supported by published data demonstrating that PAX5 is necessary for *Igk* rearrangements at the early B-cell precursor stage [45]. Since both kappa alleles are rearranged before recombination is initiated at the lambda locus [46], it can be assumed that in *Pax5*^±^ mice, a higher number of pre-BII cells undergo multiple rounds of V(D)J-recombination. With RAG enzymes being active during recombination this in turn increases the chance for off-target effects leading to malignant transformation [47, 48]. Along these lines we show that BCP-ALLs of *Pax5*^±^ mice display off-target SVs like *Kmt2c*, *Runx1* and *Phf6*. These genes share function in chromatin remodeling, DNA repair and have been found as early events predisposing to infant, T- and myeloid leukemia [49–52]. Interestingly, *Kmt2c* alterations were identified recurrently in all three leukemias. KMT2C is known to modify mono-methylation of histone H3 lysine 4 (H3K4) at enhancers and plays a role as a haploinsufficient tumor suppressor [53, 54]. Additionally, its loss is implicated in enhanced hematopoietic stem cell self-renewal capacities [49].

However, transplantation of *Pax5*^±^ pre-BII cells was not sufficient to initiate leukemia development, likely due to limited size of the persisting cell population that in part differentiated into mature B-cells, before additional potentially RAG-mediated somatic mutations could take place. Thus, these data underline the need for a differentiation arrest to enable the acquisition of secondary driver mutations. Our analysis of a c-KIT^+^ CD25^+^ cell population identified in the BM of an ageing *Pax5*^±^ cohort (termed pre-leukemia) supports this assumption, since these cells had already started to lose their B-cell identity. This was shown through analysis of TF-and-target gene sets (TF regulons), displaying elevated scores for master regulatory TFs from multiple other cell lineages, which in turn suggest a high cell plasticity - one of the hallmarks of PAX5 loss [10]. Although the amount of detected pre-leukemic clones was not sufficient to confirm additional *Pax5* alterations on a genomic level, scRNA-Seq revealed low *Pax5* expression levels coupled with *Cd19* loss. Likewise, WGS of BCP-ALLs from *Pax5*^±^ mice confirmed somatic aberrations affecting the *Pax5* locus in all samples. Thus, in the context of PAX5-driven BCP-ALL development, we postulate a PAX5-mediated differentiation block as a first step to malignant transformation, which creates an arrested susceptible precursor B-cell population in the BM. The detected skewing towards IGL in the enriched *Pax5*^±^ pre-BII population together with the maintained high levels of *Rag1* expression in the identified pre-leukemic cells suggest recurrent trials of light chain gene rearrangements in these cells predisposing them for off-target mutations. A scenario, which is further supported by the detected RAG-mediated SV in the three observed *Pax5*^±^ BCP-ALLs. These additional SV together with the augmented loss of *Pax5* and a failure to properly assemble the BCR and undergo cellular maturation leads to a concomitant de-differentiation to a pre-pro-B-like state. At this stage, the cells have a higher IL7-dependency and downregulate their *Rag1* expression. This aberrant dependency on IL-7 subsequently results in additional activating somatic mutations in the JAK-STAT (*Jak1/3* [20], *Sh2b3* [55]) and NRAS signaling pathways, as detected in our genomic analyses of *Pax5*^±^ BCP-ALL.

Interestingly, both pre-leukemic and BCP-ALL cells from the *Pax5*^±^ mouse model displayed high *Myc* levels. It was previously demonstrated that PAX5 acts in a functional loop together with EBF1 and MYC to control pro-B cell expansion through direct binding at the distal enhancers [28]. In this regard, PAX5 was shown to function as a negative regulator of MYC expression in normal B-cell progenitors [28, 56]. Thus, our data, which confirm high *Myc* levels in both pre-leukemic and leukemic cells, and increasing chromatin access at multiple Myc enhancer regions during the leukemic transformation, validate this feedback loop in an independent in vivo model with spontaneous leukemia development. MYC is well known as a key regulator of cell proliferation and thus highly relevant in this context. Together, our data suggest that low PAX5 and consequently high MYC levels play an integral role at early stages of malignant transformation. Monitoring their expression may have utility as a clinical biomarker in susceptible families and patients with a high risk of BCP-ALL progression through somatic acquisition of *Pax5* loss. *Pax5* expression level drives CD19 expression and defines the therapeutic window of potential anti-CD19 targeted treatments at early disease stages. Moreover, MYC as a therapeutic target to eradicate pre-leukemic clones has the potential to be investigated further in future studies.

## Materials and methods

### Ethics approval and consent to participate

The study was conducted in accordance with the principles of the Declaration of Helsinki. Ethical approval was obtained from the Institutional Review Boards of Tokyo Medical and Dental University (Approval No. G2000-103) and the Ethics Committee of Heinrich Heine University, Düsseldorf, Germany (Ethics Vote Number: 4886 R; Study Registration Number: 2014112933). Written informed consent for participation and publication of results was obtained from all enrolled patients and/or their legal guardians.

### Mice

B6;129S2(CBB6F)-Pax5tm1Mbu/J (*Pax5*^±^) mice were a gift from M. Busslinger. All experiments were performed with *Pax5*^±^ mice freshly backcrossed 10 times to the C57BL/6 J background. Accordingly, littermates C57BL6/J served as wild-type controls. The B6.SJL-Ptprca Pepcb/BoyJ mouse strain (CD45.1^+^, hereafter referred to as CD45.1 mice) was obtained from A. Gerbaulet. CD45.1/.2 mice were generated by crossing CD45.1 mice to C57BL/6 J mice (CD45.2^+^). Both CD45.1 and CD45.1/.2 mice were utilized as recipient mice in transplantation experiments. Both male and female mice were used for experiments. Animals were housed at the Experimental Center of the Medical Faculty of Technical University Dresden (TUD) and the Zentrum für prä-klinische Forschung (ZPF) of the Technical University Munich (TUM).

All animal experiments adhered to institutional guidelines as well as to the German Law for Protection of Animals and were approved by either the Landesdirektion Dresden or Regierung von Oberbayern (TVV60-2020 and Vet_02-22-11, respectively).

### Cell preparation

WBMCs were isolated by crushing femora and tibiae with a mortar and pestle in FACS buffer containing Phosphate-buffered saline (PBS) with 2% heat inactivated FCS (Gibco, Thermo Fisher Scientific) and 2 mM EDTA (Fisher Scientific). Afterwards, the cells were filtered through a 100 µm CellTrics filter (Sysmex Europe SE). Erythrocyte lysis was performed using 500 µl ACK lysis buffer (Thermo Fisher Scientific) and an incubation time of 5 min on ice, 10 ml FACS buffer were then added to stop the erythrocyte lysis step. Cells were centrifuged and subjected to a second filtration step through a 30 µm CellTrics filter.

Peripheral blood was extracted into glass capillaries by retro-bulbar puncture. 100 µl (PBS/250U/ml Heparin) (Biochrom) were utilized to collect the PB from the glass capillary. 60 µl of this diluted PB were ery-lysed in two sequential steps at room temperature utilizing 100 µl and 180 µl ACK lysis buffer for 10 and 5 mins respectively. Finally, a last washing step with 180 µl FACS buffer was carried out.

### B220 enrichment

For B220 enrichment, cell numbers were equally adjusted and the supernatant was removed via centrifugation at 400 g for 5 min. Cell pellets were resuspended in a mixture of 10 µl of sterile mouse CD45R (B220) MicroBeads (Milteny Biotec) diluted in 90 µl cold MACS buffer per 1 × 10^7^ cells and incubated for 15 min at 4 °C. Afterwards, 2 ml MACS buffer was added and cells were pelleted via centrifugation at 400 g for 5 min. The supernatant was aspirated and cell pellets were resuspended in 500 µl MACS buffer. LS columns (Milteny Biotec) were inserted into the MACS separator (Milteny Biotec) and primed by the addition of 3 ml MACS buffer. Cell suspensions were applied to the primed LS columns and afterwards washed thrice with 3 ml MACS buffer. LS columns were removed from the MACS separator and placed on a new collection tube. 5 ml of MACS buffer were added to each column and flushed immediately by firm application of the plunger to obtain B220-enriched cell fractions. Cell numbers were assessed and afterwards pelleted via centrifugation at 400 g for 5 min and frozen at -80 °C.

### Cell culture

Primary murine bone marrow cells were magnetically enriched for B220 positive cells and subsequently cultured on Mitomycin-C treated ST-2 feeder cells in the presence of IL-7 (Peprotech) as previously described [20].

EBV LCL were cultured in RPMI medium (Thermo Fisher Scientific) supplemented with 20% heat inactivated FCS (Gibco, Thermo Fisher Scientific) and 1% Penicillin-Streptomycin (10.000 U/ml) (Gibco, Thermo Fisher Scientific). All cells were incubated at 37°C and 5% CO_2_. LCL cell lines are confirmed Mycoplasma negative, while no Mycoplasma testing was performed for primary murine bone marrow cells.

### Transplantation

Recipient mice (CD45.1 or CD45.1/.2) were irradiated with a single dose of 9 Gray using an Yxlon Maxi Shot (γ-source).

#### Short-term pre-BII transplantation (72 h)

*Pax5*^±^ or WT donor cells were extracted, stained and sorted for pre-BII as a pool of cells from 4 donors/genotype. Subsequently, 8 CD45.1 lethally irradiated recipients (4 receiving WT and 4 receiving *Pax5*^±^ pre-BII) were transplanted retro-orbitally, each with 1 × 10^5^ sorted cells.

#### Long-term pre-BII transplantation (20 months)

Donor cells were prepared analogous to the short-term setup and additionally mixed with CD45.1 support WBMCs before being transplanted into 10 CD45.1 lethally irradiated recipients (5 receiving WT and 5 receiving *Pax5*^±^ pre-BII) as described above. Each recipient received 1×10^5^ pre-BII cells (*Pax5*^±^ or WT) mixed with 5 × 10^5^ CD45.1^+^ support cells.

#### WBMCs transplantation

Donor cells were extracted (either WT or *Pax5*^±^, each a pool of 4 donor mice) and transplanted into 10 lethally irradiated CD45.1/.2 recipient mice (5 receiving WT and 5 receiving *Pax5*^±^ WBMCs) via retro-orbital injection. Each recipient was transplanted with 4 × 10^6^ *Pax5*^±^ or WT WBMCs.

### Flow cytometry analysis

Staining was performed in 96-well plates and cell counting was carried out using a MACSquant (Miltenyi) flow cytometer. Therefore, 5 × 10^6^ extracted cells were resuspended in 50 µl FACS buffer and incubated with the respective antibodies for 30 min at 4 °C. Following each staining step (either one step staining or primary and secondary staining), cells were washed twice with FACS buffer. Cells were then resuspended in a volume of 180 µl FACS buffer and analyzed using LSRFortessa (BD Biosciences) or MACSquant (Miltenyi), or analyzed/sorted using ARIA III (BD Biosciences). 20 µl Propidium iodide (BioLegend, final concentration 1 µg/ml) were added just before measurement to discriminate between viable and dead cells. Single stain controls were used to properly compensate for spill over between different fluorochromes. Fluorescence-Minus-One (FMO) controls were used to distinguish between positive and negative populations. Final data analysis was performed with the FlowJo software Version 10.8.1 (Tree Star).

#### Basel staining

To discriminate between B-cells and other lineages, the following cocktail of biotinylated antibodies against hematopoietic lineage markers was used as a primary staining: anti-CD3 (clone 145-2C11, 1:200), anti-CD11b (clone M1/70, 1:800), anti-CD11c (clone N418, 1:800), anti- Ly-6G/Ly-6C (Gr-1) (clone RB6-8C5, 1:800), anti-NK1.1 (clone PK136, 1:800), anti-Ter119 (clone TER-119, 1:400) – all from BioLegend. Unconjugated CD16/32 (clone 93, 1:200, BioLegend) was added to avoid unspecific binding.

In this case, anti-Streptavidin V500 (1:800) from BD Horizon was added to the secondary staining mix, together with the following B-cell specific markers: anti-Human/Mouse CD45R (B220) FITC (clone RA3-6B2, 1:200), anti-Mouse CD19 eFluor 450 (clone eBio1D3 (1D3), 1:400), anti-Mouse IgM APC-eFluor 780 (clone II/41, 1:100), anti-Mouse IgD PerCP-eFluor 710 (clone 11-26c, 1:800), anti-Mouse CD117 APC (clone 2B8, 1:800), anti-Mouse CD25 PE-Cyanine 7 (clone PC61.5, 1:400), anti-Mouse CD249 (BP-1) PE (clone 6C3, 1:100) - all from eBioscience (Thermo Fisher Scientific); anti-Mouse CD127 (Il-7Rα) Brilliant Violet 711 (clone A7R34, 1:200) from BioLegend.

#### Surrogate light chain staining

Biotinylated antibodies anti-IgD (clone 11-26c, 1:100) and anti-IgM (clone II/41, 1:100) both from eBioscience were added to the primary staining mix of the Basel staining. The following antibodies were employed for secondary staining: anti-Human/Mouse CD45R (B220) APC-eFluor 780 (clone RA3-6B2, 1:100), anti-Mouse CD25 PE-Cyanine 7 (clone PC61.5, 1:400) and anti-Mouse CD19 FITC (clone MB19-1, 1:200) – all from eBioscience; anti-Mouse CD179b Brilliant Violet 421 (clone LM34, 1:50) from BDBiosciences; anti-Mouse CD179a (VpreB) PE (clone R3, 1:50) and anti-Mouse CD127 (Il-7Rα) Brilliant Violet 711 (clone A7R34, 1:200) from BioLegend; anti-Streptavidin V500 (1:800) from BD Horizon.

#### Light chain staining (Mouse)

Anti-Mouse Ig light chain κ FITC (clone RMK-45, 1:200), anti-Mouse Ig light chain λ PE (clone RML-42, 1:100) from BioLegend anti-Human/Mouse CD45R (B220) Alexa Fluor 700 (clone RA3-6B2, 1:100) from eBioscience replaced the respective fluorochromes in the secondary staining of the Basel staining.

#### Light chain staining (Human)

A single staining with the following antibodies was used: anti-Human CD19 PE (clone 4G7, 1:200), Anti-Human Ig lambda Light Chain APC (clone 1-115-2, 1:50) and Anti-Human Ig kappa Light Chain FITC (clone TB28-2, 1:50) all from eBioscience.

#### Leukemic cells staining

WBMCs isolated from *Pax5*^±^ WBMCs transplantation recipients suspected of leukemia development were subjected to 2 panels of single staining and analyzed using MACSquant (Miltenyi) flow cytometer.

**Panel 1** included anti-Human/Mouse CD45R (B220) FITC (clone RA3-6B2, 1:200), anti-Mouse CD19 eFluor 450 (clone eBio1D3 (1D3), 1:400), anti-Mouse IgM APC-eFluor 780 (clone II/41, 1:100), anti-Mouse CD117 APC (clone 2B8, 1:800), anti-Mouse CD25 PE-Cyanine 7 (clone PC61.5, 1:400), anti-Mouse CD3e PE (clone eBio500A2 (500A2), 1:100) - all from eBioscience (Thermo Fisher Scientific).

**Panel 2** included anti-Human/Mouse CD45R (B220) FITC (clone RA3-6B2, 1:200), anti-Mouse CD19 eFluor 450 (clone eBio1D3 (1D3), 1:400), anti-Mouse IgM APC-eFluor 780 (clone II/41, 1:100), anti-Mouse CD25 PE-Cyanine 7 (clone PC61.5, 1:400), anti-Mouse CD45.2 PE (clone 104, 1:100) - all from eBioscience (Thermo Fisher Scientific), and anti-mouse CD45.1 APC (clone A20, 1:100) from BioLegend. Panel 2 was mainly utilized to confirm the CD45.2 donor origin of the leukemic cells and rule out any possibility of leukemia development from residual CD45.1/.2 host cells.

HS/PC straining: In the primary staining the cocktail of biotinylated antibodies against hematopoietic lineage markers from the Basel staining was supplemented with anti-Mouse CD19 (clone MB19-1, 1:100) and anti-Human/Mouse CD45R (B220) (clone RA3-6B2, 1:400) both from BioLegend. The secondary staining was carried out with the following antibodies: anti-Mouse CD34 FITC (RAM34, 1:50), anti-Mouse CD135 APC (clone A2F10, 1:50), anti-Mouse CD127 PE (clone A7R34, 1:50), anti-Mouse CD16/32 Alexa Fluor 700 (clone 93, 1:100) all from Thermo Fisher Scientific, anti-Mouse CD48 Brilliant Violet 421 (clone HM48-1, 1:100) and anti-Mouse CD150 PE-Cyanine 7 (clone TC15-12F12.2, 1:200) both from BioLengend together with anti-Streptavidin V500 (1:800) from BD Horizon.

### Cell-cycle analysis

Extracted WBMCs from *Pax5*^±^ or WT mice were first incubated with the same biotinylated antibodies of the primary staining described in the Basel staining section to discriminate B-cells from other lineages. Cells were then subjected to a secondary staining mixture which included anti-Streptavidin APC/Cyanine7 (BioLegend, 1:400), anti-Mouse CD117 APC (clone 2B8, 1:800), anti-Mouse CD25 PE-Cyanine 7 (clone PC61.5, 1:400), anti-Human/Mouse CD45R (B220) Alexa Fluor 700 (clone RA3-6B2, 1:100), anti-Mouse CD19 PE (clone eBio1D3 (1D3), 1:1600), anti-Mouse IgM PerCP-eFluor 710 (clone II/41, 1:400), anti-Mouse IgD PerCP-eFluor 710 (clone 11-26c, 1:800) - all from eBioscience (Thermo Fisher Scientific). IgM and IgD were stained with the same fluorochrome to discriminate between (IgM^−^IgD^−^ B-cells) and (immature/recirculating B-cells).

After the two sequential washing steps following the secondary staining, cells were subjected to 90 µl of the Fixation/Permeabilization solution (BD Cytofix/Cytoperm, BD Biosciences) for 20 mins on ice, protected from light. This was followed by two washing steps employing 1x BD Perm/Wash buffer. Intracellular staining in 50 µl 1x BD Perm/Wash buffer containing either Ki67-FITC or Isotype control (BD Biosciences, 1:6) was performed for 1 h in the dark at room temperature. Cells were subsequently washed once with 1x BD Perm/Wash buffer and then stained for DNA content with 200 µl 1x BD Perm/Wash buffer including DAPI (1 µg/ml, BD Biosciences) for another hour at room temperature, in the dark. Analysis was performed directly afterwards using BD LSR Fortessa equipped with an UV laser (BD Biosciences).

### Murine qRT-PCR

For total RNA isolation, cell pellets of B220-enriched bone marrow cells from WT and *Pax5*^±^ animals were lysed in RLT buffer (Qiagen) supplemented with 2-mercaptoethanol and RNA extraction was performed following the manufacturer’s instructions of the RNeasy Mini Kit (Qiagen).

For cDNA synthesis, 1 µg of total RNA per sample and the QuantiTect Reverse Transcription Kit (Qiagen) was used according to the manufacturer’s instructions.

For total RNA extraction of different sort-purified B cell precursor subsets (i.e. pro-B, pre-BI, pre-BII, and immature B-cells) from WT and *Pax5*^±^ animals, cells were sorted directly into RLT lysis buffer supplemented with 1% 2-mercaptoethanol and RNA extraction was performed following the manufacturer’s instructions of the RNeasy Plus Micro Kit (Qiagen). The cDNA synthesis was performed based on the lowest available RNA amount of each cellular subtype using the QuantiTect Reverse Transcription Kit (Qiagen) according to the manufacturer’s protocol.

For total RNA extraction of (pre-)leukemic *Pax5*^±^ animals, WBMCs were enriched for B220 as described above. Afterwards, cells were lysed in RLT lysis buffer supplemented with 1% 2-mercaptoethanol and RNA extraction was performed following the manufacturer’s instructions of the AllPrep DNA/RNA Mini Kit (Qiagen). The cDNA synthesis was performed based on the lowest available RNA amount using the QuantiTect Reverse Transcription Kit (Qiagen) according to the manufacturer’s protocol.

For total RNA extraction of 2 weeks old WT and *Pax5*^±^ animals, WBMCs were enriched for B220 as described above and subsequently lysed in RLT lysis buffer supplemented with 1% 2-mercaptoethanol following the RNA extraction using the AllPrep DNA/RNA Mini Kit (Qiagen) according to the manufacturer’s protocol. The cDNA synthesis was performed based on the lowest available RNA amount using the QuantiTect Reverse Transcription Kit (Qiagen) according to the manufacturer’s protocol.

For qRT-PCR, predesigned TaqMan assays (Thermo Fisher) targeting the following genes were utilized: *mmHprt*: Mm03024075_m1 FAM-MGB; *mmTbp1*: Mm01277042_m1 FAM-MGB; *mmPax5*: Mm00435501_m1 FAM-MGB; *mmMyc*: Mm00487804_m1 FAM-MGB.

The qRT-PCR was carried out using the QuantiStudio 6 Pro cycler (Applied Biosystems) following the TaqMan Universal Master Mix II protocol (Applied Biosystems). Fold change analysis of gene expression between WT and *Pax5*^±^ samples was performed using the Design & Analysis 2.4 software (Applied Biosystems) and via calculation of the 2^(-ΔΔCT)^ method [57].

### Human qRT-PCR

For total RNA isolation, cell pellets of 4 ×10^6^ EBV LCL cells were lysed in RLT buffer (Qiagen) supplemented with 2-mercaptoethanol and RNA extraction was performed following the manufacturer’s instructions of the RNeasy Mini Kit (Qiagen). For cDNA synthesis, 2 µg of total RNA per sample and the QuantiTect Reverse Transcription Kit (Qiagen) was used according to the manufacturer’s instructions. For qRT-PCR, a GoTaq 2-Step RT-qPCR System and the following primers were utilized: GAPDH_F: AGATCCCTCCAAAATCAAGTGG; GAPDH_R: GGCAGAGATGATGACCCTTTT; ACTB_F: TCACCATGGATGATGATATCGC; ACTB_R: GAATCCTTCTGACCCATGCC; Pax5_SSP_F1: CTCGTCGTACTCCATCAGCG (WT specific); Pax5_SSP_F2: CTCGTCGTACTCCATCAGCA (3’ mutant specific: PAX5 c.547 G > A); hPax5_SSP_mRNA_R: AGTGCTGCCTCTCAAACACG. The qRT-PCR was carried out using the QuantiStudio 6 Pro cycler (Applied Biosystems) following the GoTaq 2-Step RT-qPCR System protocol (Promega). The ratio of gene expression between WT and *Pax5*^±^ samples was performed using the Design & Analysis 2.4 software (Applied Biosystems) and by calculating the ΔCt. Three technical replicates were performed for each EBV LCL.

### Protein quantification and Western Blot

For whole cell lysates, 4 × 10^6^ EBV LCL samples were lysed in RIPA buffer (50 mM Tris), 150 mM NaCl, 0.5% sodium deoxycholate, 1% Triton and 0.1% SDS 20%, supplemented with 10× PhosSTOP (PS, Roche) and 25× PIC (Protease Inhibitor Cocktail, Roche) for 30 min on ice. Protein concentration was measured via Bradford protein assay (Roti-Quant, Roth) by determining OD595nm. 20 µg of total protein were heated for 10 min at 95 °C while shaking at 350 rpm and loaded accordingly onto a BIORAD Mini-Protean TGX Gel 4–20% (Bio-Rad Laboratories). SDS-PAGE was performed for 2 h at 120 V. Protein transfer was performed using a Trans-Blot Turbo Transfer System (high-molecular-weight, BIO-RAD) onto a nitrocellulose membrane (BIO-RAD). The immunoblot was blocked in 1× TBS-T with 5% milk powder (Sigma-Aldrich) at room temperature for 1 h. After three washes with 1× TBS-T, the blot was incubated overnight at 4 °C with the following primary antibodies: PAX5 (Cell Signaling No. 8970, 1:1000) and GAPDH (Cell Signaling No. 5174, 1:2000) diluted in 5% Bovine Serum Albumin (Sigma-Aldrich). The following day, the secondary antibody was applied after three consecutive washes (Cell Signaling Anti-Rabbit IgG No. 7074, 1:3000) for 1 h in the dark, at room temperature, diluted in 5% Bovine Serum Albumin. After three consecutive washes, the blot was imaged after the application of an HRP-linked solution (SuperSignal West Pico PLUS Chemiluminescent Substrate, Thermo Fisher Scientific). Three biological replicates were performed (*n* = 3).

### Bulk RNA sequencing

#### Cell and library preparation

RNA was extracted from 1×10^5^ sorted pre-BII cells per mouse using the RNeasy Plus Micro Kit (Qiagen) according to the user manual. Samples comprised three WT animals and three *Pax5*^±^mice. RNA integrity was validated on a BioAnalyzer (Agilent). mRNA was isolated from 30 ng total RNA by poly-dT enrichment using the NEBNext Poly(A) mRNA Magnetic Isolation Module (NEB) according to the manufacturer’s instructions. Samples were then directly subjected to the workflow for strand-specific RNA-Seq library preparation (Ultra II Directional RNA Library Prep, NEB). For ligation NEB Next Adapter for Illumina of the NEB Next Multiplex Oligos for Illumina Kit were used. After ligation, adapters were depleted by an XP bead purification (Beckman Coulter) adding the beads solution in a ratio of 0.9:1 to the samples. Unique dual indexing was done during the following PCR enrichment (16 cycles) using amplification primers carrying the same sequence for i7 and i5 index (i5: AAT GAT ACG GCG ACC ACC GAG ATC TAC AC NNNNNNNN ACA TCT TTC CCT ACA CGA CGC TCT TCC GAT CT, i7: CAA GCA GAA GAC GGC ATA CGA GAT NNNNNNNN GTG ACT GGA GTT CAG ACG TGT GCT CTT CCG ATC T). After two more XP bead purifications (0.9:1), libraries were quantified using the Fragment Analyzer (Agilent).

#### Sequencing

Libraries were sequenced on an Illumina NovaSeq 6000 in 100 bp paired-end mode to a depth of >40 million fragments per library.

#### Data analysis

FastQC (http://www.bioinformatics.babraham.ac.uk/) was used to perform a basic quality control of the resulting sequencing data. Fragments were aligned to the mouse reference genome GRCm38 with support of the Ensembl 98 splice sites using the aligner gsnap (v2020-12-16) [58]. Counts per gene and sample were obtained based on the overlap of the uniquely mapped fragments with the same Ensembl annotation using featureCounts (v2.0.1) [59]. Normalization of raw fragments based on library size and testing for differential expression between the different genotypes was done with the DESeq2 R package (v1.30.1) [60]. Sample to sample Euclidean distance, Pearson’ and Spearman correlation coefficient (r) and PCA based upon the top 500 genes showing highest variance were computed to explore correlation between biological replicates and different libraries. To identify differentially expressed genes, counts were fitted to the negative binomial distribution and genes were tested between conditions using the Wald test of DESeq2. Resulting *p*-values were corrected for multiple testing with the Independent Hypothesis Weighting package (IHW 1.18.0) [61, 62]. Genes with a maximum of 2% false discovery rate (padj ≤ 0.02) were considered as significantly differentially expressed. Up- and downregulated genes were analyzed for enriched GO terms using the online tool Enrichr [63].

### Single-cell RNA-sequencing

#### Cell and library preparation

For single-cell analysis, TotalSeq-C mouse Hashtag antibody-DNA conjugates (BioLegend) that contain a unique sequence barcode, were added to the secondary staining mix in a 1:60 dilution to allow for pooling samples from different mice into a single sequencing run. The cell suspension was incubated for 30 min at 4 °C to allow for antibody binding, followed by four washes to remove unbound antibody. The cell pellet was resuspended in 200 µl FACS buffer. Cell sorting for the desired precursor B-cell population was carried out on an ARIA III. A total of 20,000 cells was sorted, consisting of a maximum pool of 6 conditions (= 6 hashtags). Next, the cells were loaded into the Chromium Controller microfluidics chip (10x Genomics). ScRNA-Seq was performed using the 10X Genomics Chromium technology, according to the Chromium Single-Cell 5′Reagent Kits VDJ v1.1 including BCR and user guide: CG000186_ChromiumSingleCellV_D_J_ReagentKit_FeatureBarcodingtechnology_RevA. ScRNA-seq libraries were generated with Chromium Next GEM Single-Cell 5’ Library & Gel Bead Kit v1.1, Chromium Single-Cell 5’ Library Construction Kit, Chromium Single-Cell 5’ Feature Barcode Library Kit and Chromium Single-Cell V(D)J Enrichment Kit, Mouse B Cell.

#### Sequencing

Libraries were sequenced using the NovaSeq 6000 platform (Illumina) to a depth of up to 50,000 reads for RNA and 5000 reads for V(D)J assay, with read lengths of 26 (read 1) + 8 (i7 index) + 0 (i5 index) + 91 (read 2). Raw reads were aligned to the mouse genome (mm10, refdata-cellranger-mm10-3.0.0 and refdata-cellranger-vdj-GRCm38-alts-ensembl-3.1.0) using Cell Ranger (count pipeline) (v.3.1.0).

### Data de-multiplexing by hashtag signals

The sampleIDs were recovered based on scaled hashtag antibody counts, calculated using the DSB R library v.1.0.3. The background signal level was estimated from empty droplets (selection criteria: RNA and protein count >1 and not assigned cell by Cellranger and droplet RNA and protein counts differ from respective median in this subset by median absolute deviance <2, mitochondrial read proportion <0.25) using the function DSBNormalizeProtein (denoise=F, use.isotype.control=F). Cells with at least one hashtag detected were clustered using k-means algorithm (nstarts: 100; cluster number: Nhashtag + possible pair-wise combination number +1) by these scaled hashtag antibody signals and the cluster with lowest signal used to fit a normal distribution and the 99th percentile value as cut-off (requiring that value is within range 3–5) for hashtag positivity. This cut-off was adjusted to range 3–5. Cells with signal level above this background signal level cutoff were assigned positive to the given hashtag. Cells that were annotated as individual cells (passing cutoff for only one hashtag) were used for downstream analysis.

### scRNA-Seq quality filtering and normalization

To check the quality of the libraries generated, we followed a basic QC and filtering workflow using a Python-based tools (scanpy 1.5.1, anndata 0.7.3, umap 0.4.6, numpy 1.18.5, scipy 1.5.0, pandas 1.0.5, scikit-learn 0.23.1, statsmodels 0.11.1, python-igraph 0.8.2, louvain = =0.6.1, leidenalg 0.8.1, pyvdj pyvdj 0.1.4 https://github.com/msipola/pyVDJ). Transcripts were filtered to include those that were present in more than 10 cells. To assess the viability of the cells, we quantified mitochondrial and ribosomal genes. Good quality cells were selected according to the following criteria (range used across different experiments given for cutoffs): mitochondrial gene expression percentage max 0.5–1%, number of counts min 500–5000 and max 15,000-30,000, number of expressed genes min 400–1500 and max 3000–6000.

Expression data was normalized with size factor values derived from data normalization using the scran R package v.1.26.2, and then log-transformed using the function scanpy.pp.log1p. To integrate different experiments, combat algorithm was used to remove batch effects. Highly variable genes were calculated with scanpy.pp.highly_variable_genes, selecting the top 3000 genes for principal component (PC) analysis, neighborhood graph calculation (N neighbors 15 with 50 PCs) and dimensional reduction using UMAP. Leiden clustering at different resolutions were performed to define similar transcriptome states. Cell type annotations were assigned based on hashtags and marker genes and filtered contig annotations generated by Cellranger for VDJ data were added to the data analysis. Clusters were inspected for representation of cells across donor animals, hashtags and quality control metrics. In the integrated pre-B-II data, two clusters that had unusual quality metrics and cells with mixed phenotype based on hashtag-based cell differentiation staging were suspected to represent remaining low quality cells (housekeeping ribosomal gene fraction <0.1) and therefore removed. For comparison, the variable gene selection, dimensionality reduction and clustering was then repeated for each hashtag-based cell sort separately.

### Differential expression analysis using scDD

To analyze expression changes based on the fraction of cells expressing a certain gene (DZ category) and among expressing cells by comparing the expression level and modality of distribution (DE, DP and DM categories), we used an R-based workflow (R version 4.0.3, Seurat v.4.0.1, scDD v.1.14, dittoSeq). First, normalized log2 counts based on vst transformation available in Seurat were calculated. Similar as in our previous study [64], a small pseudocount from interval (-0.1, 0.1) was added to avoid numerical instabilities. Transcripts with adj. *p*-value < 0.05 (Benjamini-Hochberg FDR method) were considered as significant. Dot plot heatmaps were used to visualize gene expression distributions, where the dot size indicates the percentage of cell group expressing each gene and color tones the average expression level.

### Differential abundance analysis

Embedding density of cells assigned to WT and mutant groups was calculated for the UMAP representation using Scanpy package. Differential abundance of cells per cluster was analyzed using Chi squared test. Cell assigned to cluster (yes/no) and genotype (WT/mutant) were used as grouping variables.

### Tabula Muris Senis reference analysis

Tabula Muris Senis bone marrow data (Marrow_droplet.h5ad) was retrieved and a subset of progenitor and B-lineage cells was selected based on provided annotation (12_hematopoietic precursor cell’, ‘18_hematopoietic precursor cell’, ‘21_late pro B cell’, ‘14_pre B cell’, ‘17_immature B cell’). The data was processed using the same Python workflow as described above to cluster cells. To compare to sorted cell populations from WT mice, label transfer analysis was performed using R Seurat package findTransferAnchors and TransferData functions based on 30 PCs. Since our sorting did not include progenitors, marker genes were used to select the clusters that connected to the pro-B cell state cells from cells assigned in TMS into ‘12_hematopoietic precursor cell’, ‘18_hematopoietic precursor cell’, which were selected and annotated as HSPC and lymphoid progenitor. The pro-B, pre-B and immature B-cell annotated matched well and together with cell-cycle phase gene set scoring were used to label clusters in non-cycling (G1) and cycling (S/G2/M) pro-B, pre-B (matched to pre-BI and pre-BII sorted cells) and immature B-cells.

### Regulon discovery and transcription factor activity scoring

TF activities that characterize specific differentiation states of WT bone marrow B-lineage were analyzed using the Tabula Muris Senis reference subset defined above and a SCENIC workflow that predicts TF activity based on TF and target gene set expression. In our implementation, equal amounts of cells per cell type were sampled from clusters defined from Tabula Muris Senis B-lineage clusters to ascertain that differences in cell type abundances do not bias the analysis. A small pseudocount (from -0.01 to 0.01 range) was used to analyze correlation to TF expression across all cells. The discovered regulons obtained from 10 runs of train-test (70:30 ratio) splits were evaluated based on left-out test set. The mean regulon scores across cell types were compared with Pearson’s product moment correlation coefficient. Regulons with *p*-value > 0.001 were discarded. The final set of regulons was then scored using the whole original data set. For filtering regulons, a linear model was fit 100 times per regulon to a subset of the regulon score matrix where 500 cells per cell type were sampled randomly from the original data set. In the model, the response is the regulon score and the cell type label is the independent variable (score ~ cell type). Regulons with the mean coefficient of determination (R^2^) < 0.5 were filtered out. Their respective scores in the WT and pre-leukemic cells were calculated and visualized as a heatmap.

### Bulk ATAC sequencing

200,000 cells of each cell population analyzed were sort-purified and frozen in 90% FCS and 10% DMSO. Cryopreserved cells were sent to Active Motif to perform ATAC-seq. The cells were thawed in a 37 °C water bath, slowly diluted with RPMI media, pelleted, washed with cold PBS, and tagmented with a Tn5 enzyme as previously described [65], with some modifications based on [66]. Briefly, cell pellets were resuspended in lysis buffer, pelleted, and tagmented using the enzyme and buffer provided in the ATAC-Seq Kit (Active Motif). Tagmented DNA was then purified using the MinElute PCR purification kit (Qiagen), amplified with 10 cycles of PCR, and the resultant libraries were purified using Agencourt AMPure SPRI beads (Beckman Coulter). Resulting material was quantified using Qubit (Invitrogen) and TapeStation (Agilent). Final libraries were sequenced with PE50 sequencing on the Illumina platform.

### Bulk ATAC data analysis

#### Pre-processing

Raw ATAC-seq data were processed using the nf-core/atacseq pipeline (v2.1.2, https://nf-co.re/atacseq/2.1.2/). Quality control was performed with FastQC to assess metrics such as per-base quality scores, sequence content, and adapter contamination. Adapter trimming was automated with Trim Galore to excise unwanted sequences. Trimmed paired-end reads were aligned to the primary reference genome using the BWA algorithm (mem mode; default settings). Duplicate reads were removed, only reads mapping as matched pairs and only uniquely mapped reads (mapping quality >= 1) were used for further analysis.

#### Peak calling

Open chromatin regions were identified using MACS2, a tool that detects significantly enriched read counts indicative of accessible genomic regions. Both narrow peaks and broad peaks (using the –broad option) were called, ensuring that regulatory elements were accurately mapped. These peak regions serve as the foundation for downstream analyses, including motif discovery and the assessment of chromatin dynamics.

#### Differential accessibility

A consensus peak count matrix was generated with each row corresponding to a peak region and each column to a sample. Differential accessibility analysis was then performed using DESeq2, which internally normalizes for library size via the median-of-ratios method. Minimal pre-filtering (retaining only peaks with at least 10 reads) and independent filtering were applied to enhance detection power. Peaks with an adjusted *p*-value below 0.05 were considered significantly differential, providing insights into the chromatin accessibility changes between experimental conditions.

#### Differential motif activity

Differential motif activity was evaluated to uncover regulatory factors underlying changes in chromatin accessibility between conditions. Using HOMER, we performed two complementary analyses: one based on the top 1000 peaks per sample (using qValue ranking) and another focusing on differentially accessible regions from pairwise comparisons. This dual approach allowed us to both scan for known transcription factor binding sites and discover novel motifs that are significantly enriched or depleted, thereby highlighting potential drivers of the observed chromatin dynamics.

The enrichment statistics (-10 log *p*-value and percentage of peaks with motif) for selected top motifs were visualized with dot plots (R package ggplot2_3.3.5). To visualize signal tracks at genomic regions of interest, the aligned BAM files (mm10 genome version) were converted to Homer tag directories (makeTagDirectory -fragLength 200, Homer/4.11 and samtools/1.12). Tag directories were then used to generate genome browser visualization files with makeMultiWigHub.pl.

### Re-analysis of bulk CUT & RUN data

To analyze PAX5 and EBF1 binding in pre-B and proB cells we used existing data from *Fedl* et al., *Nat Immunol 2024*. The bigwig signal tracks were retrieved from NCBI GEO database (GSE259358) for displaying the data as originally processed at gene loci of interest (IGV, version 2.14). To compare the ATAC-seq signal at peak locations, we retrieved the raw sequencing reads from NCBI SRA database and carried out pre-processing, alignment to mouse genome version mm10, and peak calling using Search with default settings of the nf-core CUT&RUN pipeline (nf-core/cutandrun v3.2.2, 10.5281/zenodo.5653535). Subsequently, the PAX5 peaks were motif-centered (motif GM12878_Pax5) and the signal histogram calculated within +/- 1 kb from the center using Homer (v4.11).

### Whole genome sequencing

#### DNA extraction

DNA was extracted from either tumor cells or germline tissue (mouse tail) using the AllPrep DNA/RNA mini Kit (Qiagen), according to the user manual.

#### Library preparation and sequencing

Whole genome sequencing libraries were prepared by Macrogen using the TruSeq Nano DNA Kit (Illumina) with the TruSeq Nano DNA Sample Preparation Guide, Part # 15041110 Rev. D Library Protocol. Sequencing was performed on a NovaSeq 6000 (151 bp paired-end). Tumor and germline DNA were sequenced with a minimum read depth of 60x (220Gbp) and 30x (110Gbp) respectively with an overall GC content of 41.5% and a Q30 of 91.8%.

#### Alignment, annotation and variant calling

Bioinformatic analysis of whole-genome sequencing data was done using the nf-core/Sarek workflow (software versions: Sarek 3.2.1, Python 3.10.6, Nextflow 23.04.1, Samtools 1.16.1, bwa 0.7.17-r1188 aligment with bwa mem, Fastp 0.23.2, Fastqc 0.11.9, Gawk 5.1.0, yaml 6.0, gatk4 4.3.0.0, mosdepth 0.3.3, tabix 1.12, Bcftools 1.16, Vcftools 0.1.16, CNVkit 0.9.9, Manta: 1.6.0, SVDB: 2.6.1, Tiddit: 3.3.2, Strelka: 2.9.10, ensemblvep: 106.1) using the GRCm38 reference genome. Data was analysed as tumor-normal pairs, using the same WT mouse as control sample. CNV results were retrieved from CNVkit, SVs from Manta and SNVs/indels from Strelka and Mutect2.

#### Variant filtering

Default filters were used for Strelka. Mutect2 variants fulfilling the following technical requirements were passed on to further downstream analysis: NALOD (Negative log 10 odds of artifact in normal with same allele fraction as tumor) >0, TLOD (Log 10 likelihood ratio score of variant existing versus not existing) >=10, allele fraction in tumor >=0.05 and/or 3 or more reads supporting the variant allele, no read evidence in the WT sample, no evident strand bias (i.e., evidence not detected solely from + or – strand), median base quality of alternative allele >30, and median mapping quality of alternative allele >=60. A union of the filtered variant lists from both callers constituted the list of SNVs and indels used in downstream analyses. The Ensembl VEP annotations were used to select alterations with a predicted high or moderate impact on coding regions, i.e., coding variants.

The SVs were required to pass the default Manta filters and to have both paired and split read evidence. In addition, only variants with at least 5 paired and/or split reads supporting the alternative allele in the tumor sample were included in downstream analyses. Genes affected by the SVs were determined based on Ensembl VEP annotations. CNVs were separated into events affecting autosomes and X/Y. Only events annotated with resulting copy number of either 0 (deletion of both copies), 1 (deletion of 1 copy) or >2 (duplications) were included in further analyses, also requiring the CNV events to be >=10000 bp size. Affected genes were retrieved by intersecting against GRCm38 Refseq genes (from UCSC).

Recurrence of variants was defined separately for each variant type and in addition for large deletions and duplications based on both SV and CNV calls. Oncoprint heatmap was generated with ComplexHeatmap R package. Lists of previously described BCP-ALL driver genes [67], genes from Stat/Erk pathways [68] and the discovered recurrent variants were used to define genes to show with their respective variants.

#### RSS motif annotation

To annotate RSS motifs nearby SV breakends, the breakend regions of filtered somatic SVs were extended by 100 bp up- and downstream and these extended regions were used as an input for the Meme suite FIMO tool (https://meme-suite.org/meme/tools/fimo) to scan for the consensus RSS motifs (CACAGTG, ACAAAAACC) as well as the top 237 motifs from the Supplementary Dataset 1 from Hoolehan et al. (2022) [69] (all included motif sequences are listed in Supplementary Table 7). The reference utilized in the FIMO analyses was UCSC Mammal Genomes Mouse mm10, otherwise the default parameters were used. The results were filtered to include only exact sequence matches, and the corresponding Manta SVs of each breakend with a matched RSS motif sequence were determined, including the genes annotated by VEP to be affected by the SV.

### Statistics and programs

For the statistical analyses of population frequencies, unpaired two-tailed Student’s t-tests were performed using the GraphPad Prism Software. Differences with a *p*-value < 0.05 were considered to be significant; ns = *p* > 0.05, *p ≤ 0.05, **p ≤ 0.01, ***p ≤ 0.001, ****p ≤ 0.0001.

The statistical analysis tools for genomics data are described in the respective Methods text. The *p*-values were adjusted in genomics analyses for multiple hypothesis testing using the Benjamini-Hochberg method.

Venn-Diagrams were generated with DeepVenn [70]. Genomics data was visualized with dedicated R and Python analysis packages. Illustrations were created with BioRender.com.

### Grant support

**F.A**. is supported by the Deutsche Forschungsgemeinschaft (DFG, German Research Foundation) – project number 547295412 and by EPPERMED2024-643 - GEPARD-2. **J.H**. is supported by ERC Stg 85222 “PreventALL” and Deutsche Krebshilfe (DKH) Excellenz Förderprorgamm für etablierte Wissenschaftlerinnen und Wissenschaftler 70114539. **M.H**. and **J.H**. were supported by ERA Per Med.JTC 2018 “GEPARD”. **J.H**. and **A.B**. were supported by DJCLS07R / 2019. **M.H**., **M.S**., **M.L**. and **S.M**. are supported by the Finnish Cancer Foundation, Sigrid Juselius Foundation, Academy of Finland (341693) and Jane and Aatos Erkko Foundation. **A.V**. was supported by Väre Foundation for Pediatric Cancer Research. **A.A.P**. is supported by the German Childhood Cancer Foundation (A2023/31), by the German José Carreras Foundation, Munich, Germany (DJCLS 18 R/2021) and the DZIF (TTU 07-711). **A.A.P**. and **A.B**. are supported by the German Ministry for Education and Research (Bundesministerium für Bildung und Forschung BMBF) - grant no. 01KD2410A (EDI-4-ALL), by the German Federal Office for Radiation Protection (BfS), München-Neuherberg and by the Düsseldorf School of Oncology (DSO). **A.B**. is supported by the Katharina-Hardt-Stiftung.

## Supplementary information

Supplementary Figures

Supplementary Table 1

Supplementary Table 2

Supplementary Table 3

Supplementary Table 4

Supplementary Table 5

Supplementary Table 6

Supplementary Table 7

## Data Availability

The RNA-sequencing and ATAC-sequencing data generated during and/or analyzed during the current study were uploaded to NCBI’s Gene Expression Omnibus [71] and are accessible through GEO Series accession number GSE236408 (https://www.ncbi.nlm.nih.gov/geo/query/acc.cgi?acc=GSE236408), including the bulk RNA-Sequencing data in GSE236406 (https://www.ncbi.nlm.nih.gov/geo/query/acc.cgi?acc=GSE236406), the scRNA-Sequencing data in GSE236407 (https://www.ncbi.nlm.nih.gov/geo/query/acc.cgi?acc=GSE236407) and the ATAC-Sequencing data in GSE290658 (https://www.ncbi.nlm.nih.gov/geo/query/acc.cgi?acc=GSE290658). The mouse WGS data generated here were deposited in the NCBI Sequence Read Archive (SRA) and are accessible through BioProject accession number PRJNA1066073 (https://www.ncbi.nlm.nih.gov/bioproject/PRJNA1066073). Other data are available from the corresponding authors on reasonable request.

## References

[1] Cobaleda C, Schebesta A, Delogu A, Busslinger M. Pax5: the guardian of B cell identity and function. Nat Immunol. 2007;8:463–70. 17440452 10.1038/ni1454

[2] Nutt SL, Heavey B, Rolink AG, Busslinger M. Commitment to the B-lymphoid lineage depends on the transcription factor Pax5. Nature. 1999;401:556–62. 10524622 10.1038/44076

[3] McManus S, Ebert A, Salvagiotto G, Medvedovic J, Sun Q, Tamir I, et al. The transcription factor Pax5 regulates its target genes by recruiting chromatin-modifying proteins in committed B cells. EMBO J. 2011;30:2388–404. 21552207 10.1038/emboj.2011.140 PMC3116275

[4] Fedl AS, Tagoh H, Gruenbacher S, Sun Q, Schenk RL, Froussios K, et al. Transcriptional function of E2A, Ebf1, Pax5, Ikaros and Aiolos analyzed by in vivo acute protein degradation in early B cell development. Nat Immunol. 2024;25:1663–77. 39179932 10.1038/s41590-024-01933-7

[5] Revilla IDR, Bilic I, Vilagos B, Tagoh H, Ebert A, Tamir IM, et al. The B-cell identity factor Pax5 regulates distinct transcriptional programmes in early and late B lymphopoiesis. EMBO J. 2012;31:3130–46. 22669466 10.1038/emboj.2012.155 PMC3400013

[6] Pridans C, Holmes ML, Polli M, Wettenhall JM, Dakic A, Corcoran LM, et al. Identification of Pax5 target genes in early B cell differentiation. J Immunol. 2008;180:1719–28. 18209069 10.4049/jimmunol.180.3.1719

[7] Chan LN, Chen Z, Braas D, Lee JW, Xiao G, Geng H, et al. Metabolic gatekeeper function of B-lymphoid transcription factors. Nature. 2017;542:479–83. 28192788 10.1038/nature21076 PMC5621518

[8] Schebesta A, McManus S, Salvagiotto G, Delogu A, Busslinger GA, Busslinger M. Transcription factor Pax5 activates the chromatin of key genes involved in B cell signaling, adhesion, migration, and immune function. Immunity. 2007;27:49–63. 17658281 10.1016/j.immuni.2007.05.019

[9] Urbanek P, Wang ZQ, Fetka I, Wagner EF, Busslinger M. Complete block of early B cell differentiation and altered patterning of the posterior midbrain in mice lacking Pax5/BSAP. Cell. 1994;79:901–12. 8001127 10.1016/0092-8674(94)90079-5

[10] Cobaleda C, Jochum W, Busslinger M. Conversion of mature B cells into T cells by dedifferentiation to uncommitted progenitors. Nature. 2007;449:473–7. 17851532 10.1038/nature06159

[11] Rolink AG, Schaniel C, Bruno L, Melchers F. In vitro and in vivo plasticity of Pax5-deficient pre-B I cells. Immunol Lett. 2002;82:35–40. 12008032 10.1016/s0165-2478(02)00016-0

[12] Mullighan CG, Goorha S, Radtke I, Miller CB, Coustan-Smith E, Dalton JD, et al. Genome-wide analysis of genetic alterations in acute lymphoblastic leukaemia. Nature. 2007;446:758–64. 17344859 10.1038/nature05690

[13] Gu Z, Churchman ML, Roberts KG, Moore I, Zhou X, Nakitandwe J, et al. PAX5-driven subtypes of B-progenitor acute lymphoblastic leukemia. Nat Genet. 2019;51:296–307. 30643249 10.1038/s41588-018-0315-5 PMC6525306

[14] Jia Z, Gu Z. PAX5 alterations in B-cell acute lymphoblastic leukemia. Front Oncol. 2022;12:1023606. 36387144 10.3389/fonc.2022.1023606 PMC9640836

[15] Auer F, Ruschendorf F, Gombert M, Husemann P, Ginzel S, Izraeli S, et al. Inherited susceptibility to pre B-ALL caused by germline transmission of PAX5 c.547G>A. Leukemia. 2014;28:1136–8. 24287434 10.1038/leu.2013.363

[16] Shah S, Schrader KA, Waanders E, Timms AE, Vijai J, Miething C, et al. A recurrent germline PAX5 mutation confers susceptibility to pre-B cell acute lymphoblastic leukemia. Nat Genet. 2013;45:1226–31. 24013638 10.1038/ng.2754 PMC3919799

[17] Duployez N, Jamrog LA, Fregona V, Hamelle C, Fenwarth L, Lejeune S, et al. Germline PAX5 mutation predisposes to familial B-cell precursor acute lymphoblastic leukemia. Blood. 2021;137:1424–8. 33036026 10.1182/blood.2020005756

[18] Yazdanparast S, Khatami SR, Galehdari H, Jaseb K. One missense mutation in exon 2 of the PAX5 gene in Iran. Genet Mol Res. 2015;14:17768–75. 26782422 10.4238/2015.December.22.1

[19] Escudero A, Takagi M, Auer F, Friedrich UA, Miyamoto S, Ogawa A, et al. Clinical and immunophenotypic characteristics of familial leukemia predisposition caused by PAX5 germline variants. Leukemia. 2022;36:2338–42. 35902733 10.1038/s41375-022-01661-7

[20] Martin-Lorenzo A, Hauer J, Vicente-Duenas C, Auer F, Gonzalez-Herrero I, Garcia-Ramirez I, et al. Infection Exposure is a Causal Factor in B-cell Precursor Acute Lymphoblastic Leukemia as a Result of Pax5-Inherited Susceptibility. Cancer Discov. 2015;5:1328–43. 26408659 10.1158/2159-8290.CD-15-0892

[21] Bettini LR, Fazio G, Saitta C, Piazza R, Palamini S, Buracchi C, et al. Diverse mechanisms of leukemogenesis associated with PAX5 germline mutation. Leukemia. 2024;38:2479–82. 39256601 10.1038/s41375-024-02399-0 PMC11518994

[22] Garcia-Solorio J, Martinez-Villegas O, Rodriguez-Corona U, Molina-Garay C, Jimenez-Olivares M, Carrillo-Sanchez K, et al. Case report: A familial B-acute lymphoblastic leukemia associated with a new germline pathogenic variant in PAX5. The first report in Mexico. Front Oncol. 2024;14:1355335. 38571503 10.3389/fonc.2024.1355335 PMC10987763

[23] van Engelen N, Roest M, van Dijk F, Sonneveld E, Bladergroen R, van Reijmersdal SV, et al. A novel germline PAX5 single exon deletion in a pediatric patient with precursor B-cell leukemia. Leukemia. 2023;37:1908–11. 37543654 10.1038/s41375-023-01991-0 PMC10457179

[24] Fuxa M, Busslinger M. Reporter gene insertions reveal a strictly B lymphoid-specific expression pattern of Pax5 in support of its B cell identity function. J Immunol. 2007;178:8222–8. 17600970 10.4049/jimmunol.178.12.8221-a

[25] Hardy RR, Carmack CE, Shinton SA, Kemp JD, Hayakawa K. Resolution and characterization of pro-B and pre-pro-B cell stages in normal mouse bone marrow. J Exp Med. 1991;173:1213–25. 1827140 10.1084/jem.173.5.1213 PMC2118850

[26] Rolink A, Grawunder U, Winkler TH, Karasuyama H, Melchers F. IL-2 receptor alpha chain (CD25, TAC) expression defines a crucial stage in pre-B cell development. Int Immunol. 1994;6:1257–64. 7526894 10.1093/intimm/6.8.1257

[27] Aibar S, Gonzalez-Blas CB, Moerman T, Huynh-Thu VA, Imrichova H, Hulselmans G, et al. SCENIC: single-cell regulatory network inference and clustering. Nat Methods. 2017;14:1083–6. 28991892 10.1038/nmeth.4463 PMC5937676

[28] Somasundaram R, Jensen CT, Tingvall-Gustafsson J, Ahsberg J, Okuyama K, Prasad M, et al. EBF1 and PAX5 control pro-B cell expansion via opposing regulation of the Myc gene. Blood. 2021;137:3037–49. 33619557 10.1182/blood.2020009564 PMC8176764

[29] Alsuwaidi L, Hachim M, Senok A. Novel Markers in Pediatric Acute Lymphoid Leukemia: The Role of ADAM6 in B Cell Leukemia. Front Cell Dev Biol. 2021;9:706129. 34249950 10.3389/fcell.2021.706129 PMC8269160

[30] Xu J, Liu Y, Chen C, Niu T. KMT2D Is a Haploinsufficient tumor suppressor in acute Leukemia. Blood. 2018;132:1511.

[31] Melchers F, Karasuyama H, Haasner D, Bauer S, Kudo A, Sakaguchi N, et al. The surrogate light chain in B-cell development. Immunol Today. 1993;14:60–8. 8166770 10.1016/0167-5699(93)90060-X

[32] Kudo A, Melchers F. A second gene, VpreB in the lambda 5 locus of the mouse, which appears to be selectively expressed in pre-B lymphocytes. EMBO J. 1987;6:2267–72. 3117530 10.1002/j.1460-2075.1987.tb02500.x PMC553628

[33] Sakaguchi N, Melchers F. Lambda 5, a new light-chain-related locus selectively expressed in pre-B lymphocytes. Nature. 1986;324:579–82. 3024017 10.1038/324579a0

[34] Loffert D, Ehlich A, Muller W, Rajewsky K. Surrogate light chain expression is required to establish immunoglobulin heavy chain allelic exclusion during early B cell development. Immunity. 1996;4:133–44. 8624804 10.1016/s1074-7613(00)80678-0

[35] Heltemes-Harris LM, Hubbard GK, LaRue RS, Munro SA, Yang R, Henzler CM, et al. Identification of mutations that cooperate with defects in B cell transcription factors to initiate leukemia. Oncogene. 2021;40:6166–79. 34535769 10.1038/s41388-021-02012-z PMC8556320

[36] Heltemes-Harris LM, Willette MJ, Ramsey LB, Qiu YH, Neeley ES, Zhang N, et al. Ebf1 or Pax5 haploinsufficiency synergizes with STAT5 activation to initiate acute lymphoblastic leukemia. J Exp Med. 2011;208:1135–49. 21606506 10.1084/jem.20101947 PMC3173246

[37] Martin-Lorenzo A, Auer F, Chan LN, Garcia-Ramirez I, Gonzalez-Herrero I, Rodriguez-Hernandez G, et al. Loss of Pax5 Exploits Sca1-BCR-ABL(p190) Susceptibility to Confer the Metabolic Shift Essential for pB-ALL. Cancer Res. 2018;78:2669–79. 29490943 10.1158/0008-5472.CAN-17-3262 PMC6245574

[38] Vicente-Duenas C, Janssen S, Oldenburg M, Auer F, Gonzalez-Herrero I, Casado-Garcia A, et al. An intact gut microbiome protects genetically predisposed mice against leukemia. Blood. 2020;136:2003–17. 32911536 10.1182/blood.2019004381 PMC7694022

[39] Calderon L, Schindler K, Malin SG, Schebesta A, Sun Q, Schwickert T, et al. Pax5 regulates B cell immunity by promoting PI3K signaling via PTEN down-regulation. Sci Immunol. 2021;6:eabg5003. 10.1126/sciimmunol.abg5003 PMC7611449 34301800

[40] Clark MR, Mandal M, Ochiai K, Singh H. Orchestrating B cell lymphopoiesis through interplay of IL-7 receptor and pre-B cell receptor signalling. Nat Rev Immunol. 2014;14:69–80. 24378843 10.1038/nri3570 PMC4276135

[41] Lee J, Robinson ME, Sun R, Kume K, Ma N, Cosgun KN, et al. Dynamic phosphatase-recruitment controls B-cell selection and oncogenic signaling. bioRxiv. 2023 10.1101/2023.03.13.532151.

[42] Kuppers R. B cells under influence: transformation of B cells by Epstein-Barr virus. Nat Rev Immunol. 2003;3:801–12. 14523386 10.1038/nri1201

[43] Nutt SL, Urbanek P, Rolink A, Busslinger M. Essential functions of Pax5 (BSAP) in pro-B cell development: difference between fetal and adult B lymphopoiesis and reduced V-to-DJ recombination at the IgH locus. Genes Dev. 1997;11:476–91. 9042861 10.1101/gad.11.4.476

[44] Hesslein DG, Pflugh DL, Chowdhury D, Bothwell AL, Sen R, Schatz DG. Pax5 is required for recombination of transcribed, acetylated, 5’ IgH V gene segments. Genes Dev. 2003;17:37–42. 12514097 10.1101/gad.1031403 PMC195966

[45] Sato H, Saito-Ohara F, Inazawa J, Kudo A. Pax-5 is essential for kappa sterile transcription during Ig kappa chain gene rearrangement. J Immunol. 2004;172:4858–65. 15067064 10.4049/jimmunol.172.8.4858

[46] Hieter PA, Korsmeyer SJ, Waldmann TA, Leder P. Human immunoglobulin kappa light-chain genes are deleted or rearranged in lambda-producing B cells. Nature. 1981;290:368–72. 6783958 10.1038/290368a0

[47] Geron I, Savino AM, Fishman H, Tal N, Brown J, Turati VA, et al. An instructive role for Interleukin-7 receptor alpha in the development of human B-cell precursor leukemia. Nat Commun. 2022;13:659. 35115489 10.1038/s41467-022-28218-7 PMC8814001

[48] Papaemmanuil E, Rapado I, Li Y, Potter NE, Wedge DC, Tubio J, et al. RAG-mediated recombination is the predominant driver of oncogenic rearrangement in ETV6-RUNX1 acute lymphoblastic leukemia. Nat Genet. 2014;46:116–25. 24413735 10.1038/ng.2874 PMC3960636

[49] Chen R, Okeyo-Owuor T, Patel RM, Casey EB, Cluster AS, Yang W, et al. Kmt2c mutations enhance HSC self-renewal capacity and convey a selective advantage after chemotherapy. Cell Rep. 2021;34:108751. 33596429 10.1016/j.celrep.2021.108751 PMC7951951

[50] Kurzer JH, Weinberg OK. PHF6 Mutations in Hematologic Malignancies. Front Oncol. 2021;11:704471. 34381727 10.3389/fonc.2021.704471 PMC8350393

[51] Li Y, Yang W, Devidas M, Winter SS, Kesserwan C, Yang W, et al. Germline RUNX1 variation and predisposition to childhood acute lymphoblastic leukemia. J Clin Invest. 2021;131:e147898. 10.1172/JCI147898 PMC8409579 34166225

[52] Maurya S, Yang W, Tamai M, Zhang Q, Erdmann-Gilmore P, Bystry A, et al. Loss of KMT2C reprograms the epigenomic landscape in hPSCs resulting in NODAL overexpression and a failure of hemogenic endothelium specification. Epigenetics. 2022;17:220–38. 34304711 10.1080/15592294.2021.1954780 PMC8865227

[53] Chen C, Liu Y, Rappaport AR, Kitzing T, Schultz N, Zhao Z, et al. MLL3 is a haploinsufficient 7q tumor suppressor in acute myeloid leukemia. Cancer Cell. 2014;25:652–65. 24794707 10.1016/j.ccr.2014.03.016 PMC4206212

[54] Wang LH, Aberin MAE, Wu S, Wang SP. The MLL3/4 H3K4 methyltransferase complex in establishing an active enhancer landscape. Biochem Soc Trans. 2021;49:1041–54. 34156443 10.1042/BST20191164 PMC8286814

[55] Perez-Garcia A, Ambesi-Impiombato A, Hadler M, Rigo I, LeDuc CA, Kelly K, et al. Genetic loss of SH2B3 in acute lymphoblastic leukemia. Blood. 2013;122:2425–32. 23908464 10.1182/blood-2013-05-500850 PMC3790510

[56] Ramamoorthy S, Kometani K, Herman JS, Bayer M, Boller S, Edwards-Hicks J, et al. EBF1 and Pax5 safeguard leukemic transformation by limiting IL-7 signaling, Myc expression, and folate metabolism. Genes Dev. 2020;34:1503–19. 33004416 10.1101/gad.340216.120 PMC7608749

[57] Livak KJ, Schmittgen TD. Analysis of relative gene expression data using real-time quantitative PCR and the 2(-Delta Delta C(T)) Method. Methods. 2001;25:402–8. 11846609 10.1006/meth.2001.1262

[58] Wu TD, Nacu S. Fast and SNP-tolerant detection of complex variants and splicing in short reads. Bioinformatics. 2010;26:873–81. 20147302 10.1093/bioinformatics/btq057 PMC2844994

[59] Liao Y, Smyth GK, Shi W. featureCounts: an efficient general purpose program for assigning sequence reads to genomic features. Bioinformatics. 2014;30:923–30. 24227677 10.1093/bioinformatics/btt656

[60] Love MI, Huber W, Anders S. Moderated estimation of fold change and dispersion for RNA-seq data with DESeq2. Genome Biol. 2014;15:550. 25516281 10.1186/s13059-014-0550-8 PMC4302049

[61] Ignatiadis N, Huber W. Covariate powered cross-weighted multiple testing 2017 January 01, 2017:[arXiv:1701.05179 p.]. Available from: https://ui.adsabs.harvard.edu/abs/2017arXiv170105179I.

[62] Ignatiadis N, Klaus B, Zaugg JB, Huber W. Data-driven hypothesis weighting increases detection power in genome-scale multiple testing. Nat Methods. 2016;13:577–80. 27240256 10.1038/nmeth.3885 PMC4930141

[63] Xie Z, Bailey A, Kuleshov MV, Clarke DJB, Evangelista JE, Jenkins SL, et al. Gene set knowledge discovery with enrichr. Curr Protoc. 2021;1:e90. 33780170 10.1002/cpz1.90 PMC8152575

[64] Mehtonen J, Teppo S, Lahnalampi M, Kokko A, Kaukonen R, Oksa L, et al. Single cell characterization of B-lymphoid differentiation and leukemic cell states during chemotherapy in ETV6-RUNX1-positive pediatric leukemia identifies drug-targetable transcription factor activities. Genome Med. 2020;12:99. 33218352 10.1186/s13073-020-00799-2 PMC7679990

[65] Buenrostro JD, Giresi PG, Zaba LC, Chang HY, Greenleaf WJ. Transposition of native chromatin for fast and sensitive epigenomic profiling of open chromatin, DNA-binding proteins and nucleosome position. Nat Methods. 2013;10:1213–8. 24097267 10.1038/nmeth.2688 PMC3959825

[66] Corces MR, Trevino AE, Hamilton EG, Greenside PG, Sinnott-Armstrong NA, Vesuna S, et al. An improved ATAC-seq protocol reduces background and enables interrogation of frozen tissues. Nat Methods. 2017;14:959–62. 28846090 10.1038/nmeth.4396 PMC5623106

[67] Ueno H, Yoshida K, Shiozawa Y, Nannya Y, Iijima-Yamashita Y, Kiyokawa N, et al. Landscape of driver mutations and their clinical impacts in pediatric B-cell precursor acute lymphoblastic leukemia. Blood Adv. 2020;4:5165–73. 33095873 10.1182/bloodadvances.2019001307 PMC7594377

[68] Chan LN, Murakami MA, Robinson ME, Caeser R, Sadras T, Lee J, et al. Signalling input from divergent pathways subverts B cell transformation. Nature. 2020;583:845–51. 32699415 10.1038/s41586-020-2513-4 PMC7394729

[69] Hoolehan W, Harris JC, Byrum JN, Simpson DA, Rodgers KK. An updated definition of V(D)J recombination signal sequences revealed by high-throughput recombination assays. Nucleic Acids Res. 2022;50:11696–711. 36370096 10.1093/nar/gkac1038 PMC9723617

[70] Hulsen T DeepVenn – a web application for the creation of area-proportional Venn diagrams using the deep learning framework Tensorflow.js2022 September 01, 2022:[arXiv:2210.04597 p.]. Available from: https://ui.adsabs.harvard.edu/abs/2022arXiv221004597H.

[71] Edgar R, Domrachev M, Lash AE. Gene Expression Omnibus: NCBI gene expression and hybridization array data repository. Nucleic Acids Res. 2002;30:207–10. 11752295 10.1093/nar/30.1.207 PMC99122

