# Supplementary material for: Trajectories from single-cells to PAX5-driven leukemia reveal PAX5-MYC interplay in vivo
Source: Leukemia. 2025 May 20;39(7):1607–26. doi: 10.1038/s41375-025-02626-2 (PMC12208885; doi:10.1038/s41375-025-02626-2)
Supplement: Supplementary file 1 — Supplementary Figures [file 41375_2025_2626_MOESM1_ESM.pdf]

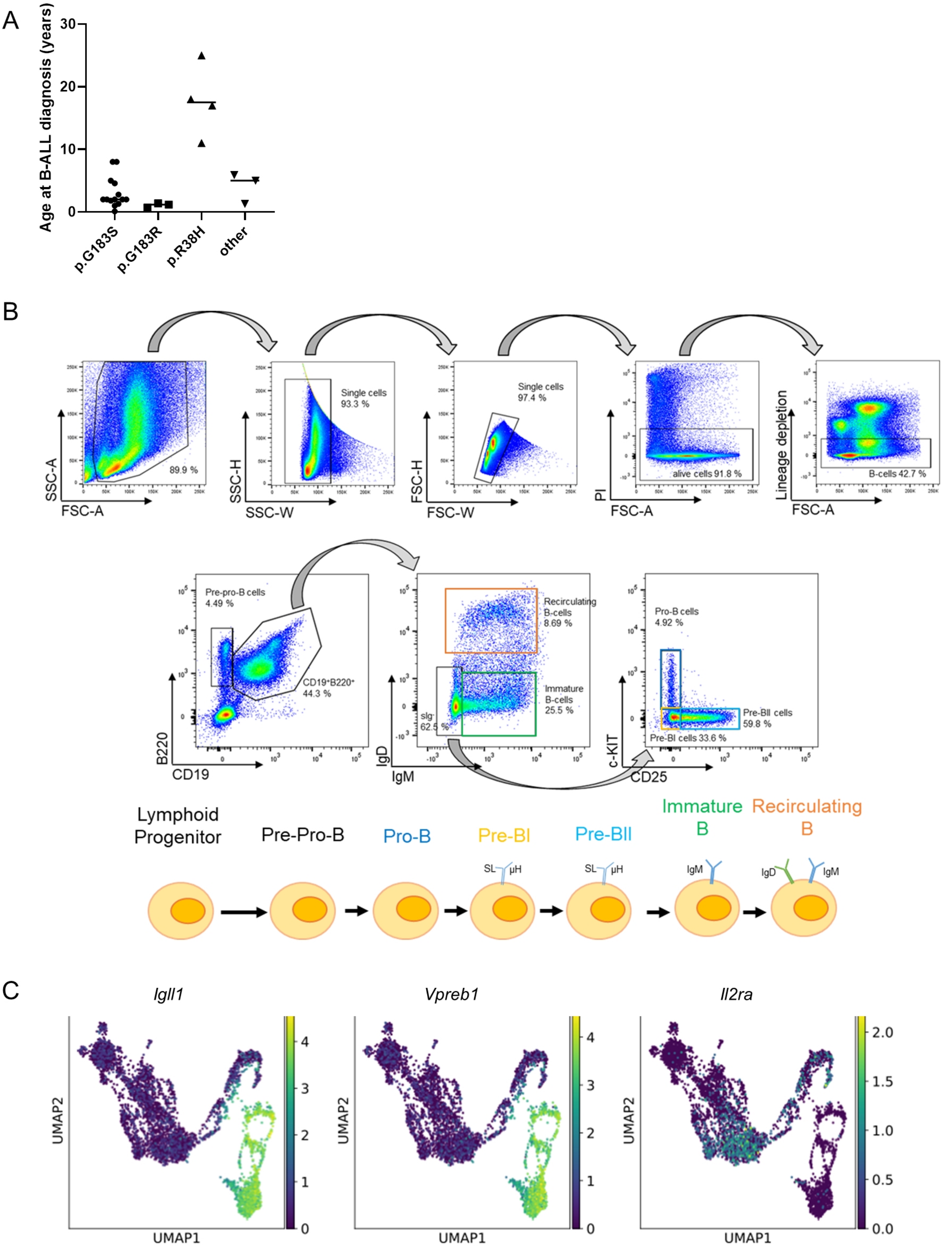

Figure S1:
A: Age of B-cell precursor acute lymphoblastic leukemia (BCP-ALL) onset for patients displayed in Figure 1A carrying different *PAX5* germline variants.
B: Representative flow cytometry plots of the employed Basel staining strategy on 11 weeks old mice (20). After doublet exclusion, viable cells are selected and all lineage committed cells that are non-B are excluded. B-cell differentiation stages are subsequently gated according to the following markers: pre-pro-B (B220$^+$ CD19$^-$), pro-B (B220$^+$ CD19$^+$ c-KIT$^+$ CD25$^-$ IgM$^-$ IgD$^-$), pre-BI (B220$^+$ CD19$^+$ c-KIT$^-$ CD25$^-$ IgM$^-$ IgD$^-$), pre-BII (B220$^+$ CD19$^+$ c-KIT$^-$ CD25$^+$ IgM$^-$ IgD$^-$), immature B (B220$^+$ CD19$^+$ IgM$^+$ IgD$^-$) and recirculating B (B220$^+$ CD19$^+$ IgM$^+$ IgD$^+$). The different precursor B-cell subsets are schematically displayed below and the respective gates colored accordingly.
C: Single cell gene expression of surrogate light chain genes *Igll1* and *Vpreb1* as well as *Il2ra* across the different sorted wild-type (WT) precursor B-cell subsets.

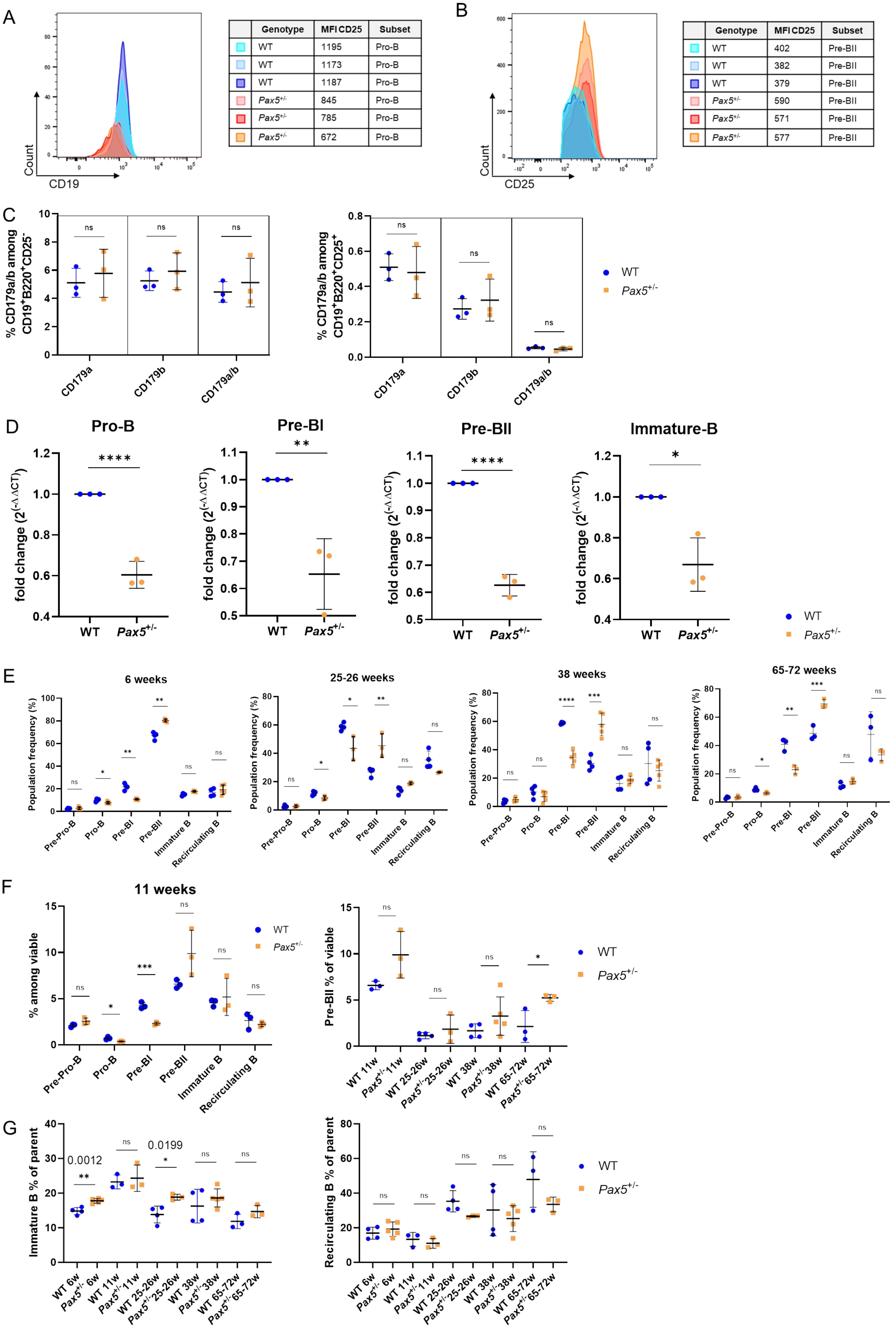
A
Count
CD19
Genotype | MFI CD25 | Subset
WT | 1195 | Pro-B
WT | 1173 | Pro-B
WT | 1187 | Pro-B
Pax5+/- | 845 | Pro-B
Pax5+/- | 785 | Pro-B
Pax5+/- | 672 | Pro-B
B
Count
CD25
Genotype | MFI CD25 | Subset
WT | 402 | Pre-BII
WT | 382 | Pre-BII
WT | 379 | Pre-BII
Pax5+/- | 590 | Pre-BII
Pax5+/- | 571 | Pre-BII
Pax5+/- | 577 | Pre-BII
C
% CD179a/b among CD19+B220+CD25-
% CD179a/b among CD19+B220+CD25+
CD179a
CD179b
CD179a/b
ns
WT
Pax5+/-
D
Pro-B
Pre-BI
Pre-BII
Immature-B
fold change (2(-ΔΔCT))
****
**
*
WT
Pax5+/-
E
6 weeks
25-26 weeks
38 weeks
65-72 weeks
Population frequency (%)
Pre-Pro-B
Pro-B
Pre-BI
Pre-BII
Immature B
Recirculating B
F
11 weeks
% among viable
Pre-BII % of viable
WT 11w
Pax5+/- 11w
WT 25-26w
Pax5+/- 25-26w
WT 38w
Pax5+/- 38w
WT 65-72w
Pax5+/- 65-72w
G
Immature B % of parent
Recirculating B % of parent
0.0012
0.0199
WT 6w
Pax5+/- 6w

H

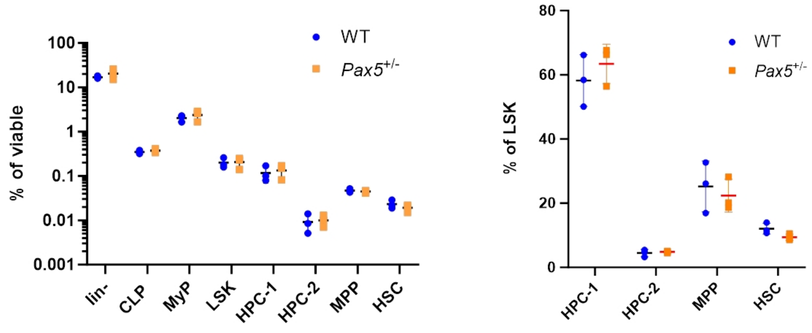

I

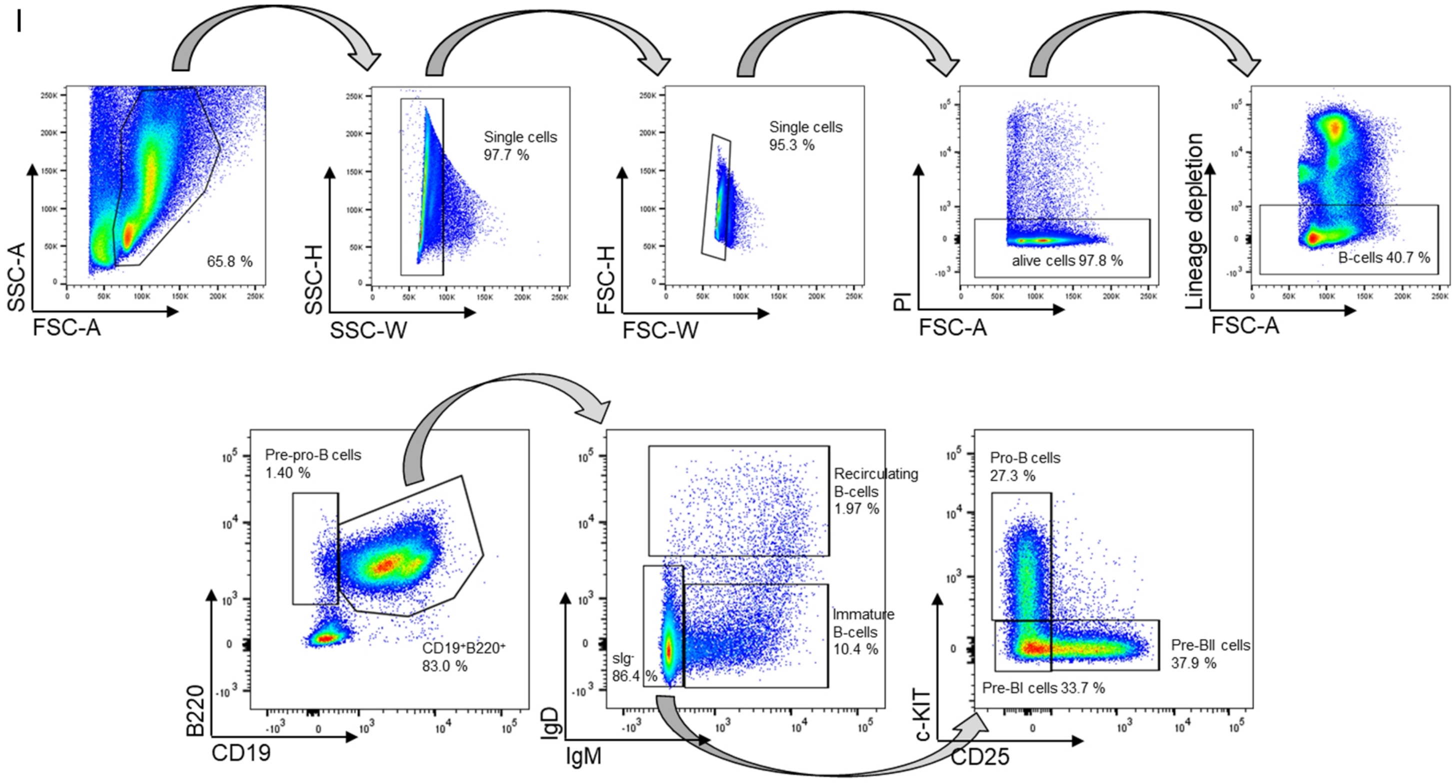

Figure S2:
A: Mean fluorescence intensity (MFI) plot and recorded MFI values of CD19 in pro-B cells of *Pax5*$^{+/-}$ mice compared to wild-type (WT) littermates (left).
B: Analogous representation to A) for CD25 MFI in pre-BII cells.
C: Expression of CD179a, CD179b or both (CD179a/b) on the cell surface of CD25^{+} and CD25^{-} B-cell precursors in the bone marrow of WT and *Pax5*$^{+/-}$ mice. No significant differences are observed comparing n=3 *Pax5*$^{+/-}$ and n=3 WT littermates 12 weeks of age.
D: qRT-PCR analysis showing *Pax5* gene expression in sort-purified B-cell subsets (pro-B, pre-BI, pre-BII and immature-B) of 6 weeks old *Pax5*$^{+/-}$ compared to WT animals (n=3 each).
E: Basel staining showing population frequencies of the different bone marrow B-cell precursor subsets between WT and *Pax5*$^{+/-}$ mice for different aging cohorts.
F: Displayed are WT and *Pax5*$^{+/-}$ bone marrow B-cell precursor subsets (n=3 for each genotype, all 11 weeks) (left) and pre-BII cells (right) of the different aging cohorts as percentage among viable cells.
G: Percentage of immature B-cells (left) and recirculating B-cells (right) recorded for different aging cohorts comparing WT and *Pax5*$^{+/-}$ mice.
H: Displayed are percentages of hematopoietic stem and progenitor cells (HS/PCs) between WT and *Pax5*$^{+/-}$ mice either among % of viable (left) or % of LSK (right). CLP: lin^{-} CD135^{+} CD127^{+}, MyP: lin^{-} Sca1^{-} c-KIT^{+}; LSK: lin^{-} Sca1^{+} c-KIT^{+}; HPC-1: LSK CD150^{-} CD48^{+}; HPC-2: LSK CD150^{+} CD48^{+}; MPP: LSK CD150^{-} CD48^{-}; HSC: LSK CD150^{+} CD48^{-}; Lin = Lineage; CLP = Common lymphoid progenitors; MyP = Myeloid progenitors; LSK = Lineage negative, Sca-1 positive, c-KIT positive; HPC-1 = Hematopoietic Progenitor Cell 1; HPC-2 = Hematopoietic Progenitor Cell 2; MPP = Multipotent progenitors; HSC = Hematopoietic stem cells.
I: Representative flow cytometry plots of the employed Basel staining on 2 weeks old mice. Recirculating B-cells are still sparse as they are still in the process of development at this early age.
Displayed are individual values with mean and SD. A two-tailed Student's unpaired t-test was performed for the statistical analysis. ns = not significant, * p ≤ 0.05, ** p ≤ 0.01, *** p ≤ 0.001, **** p ≤ 0.0001.

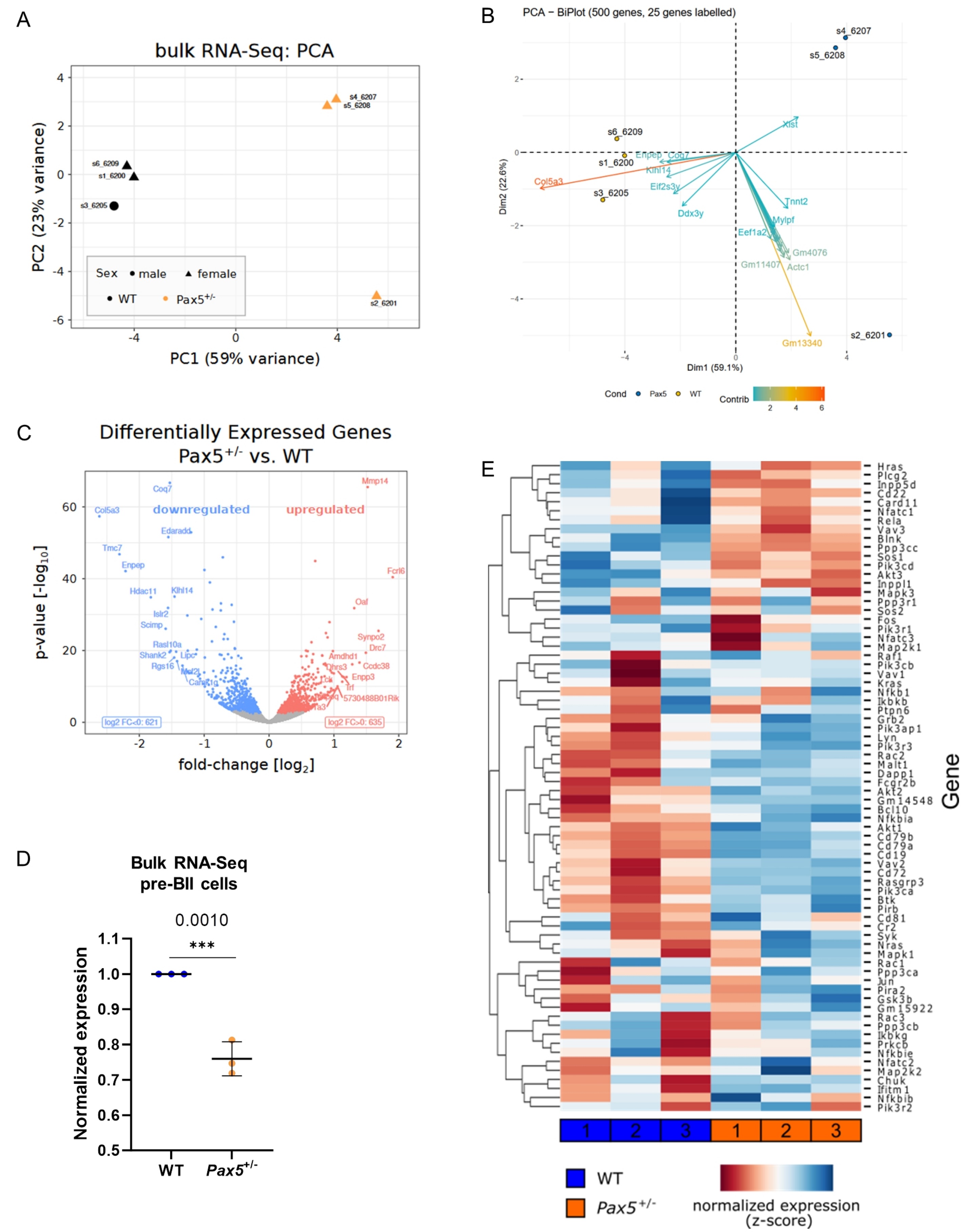

Figure S3:

A: Principal component analysis (PCA) of the bulk RNA-Sequencing (RNA-Seq) transcriptome including the top 500 most diverse genes among all 6 samples – three wild-type (WT) mouse pre-BII cells and three *Pax5*$^{+/-}$ pre-BII cells.

B: PCA biplot, showing the influence of the different genes within the PCA. The deviation of sample s2_6201 in PC2 (Figure S3A) is mostly attributed to differences in Gm13340 gene expression, which is irrelevant for our analysis.

C: Differentially expressed genes (DEGs) in *Pax5*$^{+/-}$ pre-BII cells compared to WT pre-BII are identified after correction for multiple testing with false discovery rate (FDR)=2%. 1,256 DEGs are noted, either upregulated (n=635) or downregulated (n=621).

D: *Pax5* gene expression in the bulk RNA-Seq performed for *Pax5*$^{+/-}$ pre-BII cells normalized to WT controls (see Figure 3A). A two-tailed Student's unpaired t-test was performed for the statistical analysis. *** $p \leq 0.001$.

E: Heatmap displaying gene expression differences identified in Figure 4B between WT and *Pax5*$^{+/-}$ pre-BII cells in genes related to B-cell receptor (BCR) signaling.

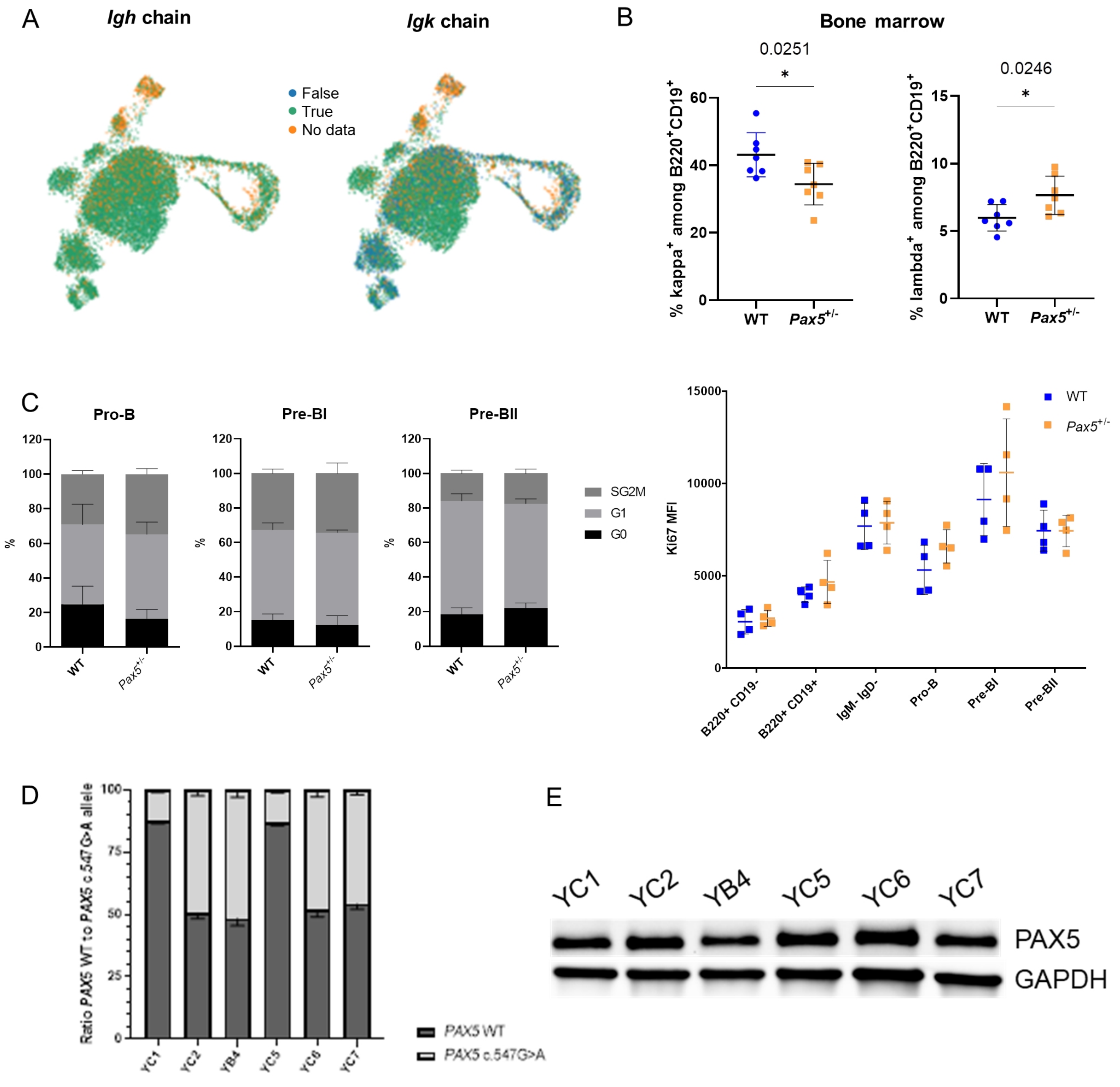

Figure S4:

A: Expressed immunoglobulin heavy chain (IGH) and immunoglobulin kappa light chain (IGK) status of pre-BII cells analyzed by single-cell RNA-sequencing. True = chain expression; False = no chain expression; No data = no chain detected.

B: Flow cytometry analysis showing a lower percentage of kappa and higher percentage of lambda light chain rearranged B-cells in the bone marrow of n=7 *Pax5*$^{+/-}$ mice vs. n=7 wild-type (WT) mice, ages 11-16 weeks. Displayed are individual values with mean and SD. A two-tailed Student's unpaired t-test was performed for the statistical analysis. Respective p-values are indicated. * $p \leq 0.05$

C: Cell-cycle analysis of B-cell progenitors from *Pax5*$^{+/-}$ vs. WT mice (n= 4 vs 4, age 10-13 weeks). Left panel displays the distribution of pro-B, pre-BI and pre-BII cells from *Pax5*$^{+/-}$ and WT mice among the different cell-cycle stages (G0, G1, S/G2/M). Right panel shows the median fluorescence intensity of Ki-67 expression among different subsets of B-cell progenitors isolated from the bone marrow of both mouse groups. No significant difference in cell-cycle was reported for any of the analyzed B-cell progenitor populations between *Pax5*$^{+/-}$ and littermate controls.

D: Allele specific qRT-PCR for *PAX5*, detecting either *PAX5* c.547G (WT) or *PAX5* c.547A (*PAX5* p.G183S variant). YC1 and YC5 are WT controls. Small mutant detection levels in YC1 and YC5 are due to un-specific bindings of the mutant primer. The ratios were normalized to 100%.

E: Western Blot analysis for PAX5 protein expression in Epstein-Barr Virus (EBV)-transformed lymphoblastoid cell lines from individuals carrying WT PAX5 (YC1 and YC5) or germline variants of PAX5 (heterozygous PAX5 p.G183S, YC2, YB4, YC6, YC7). GAPDH served as general loading control.

A

B

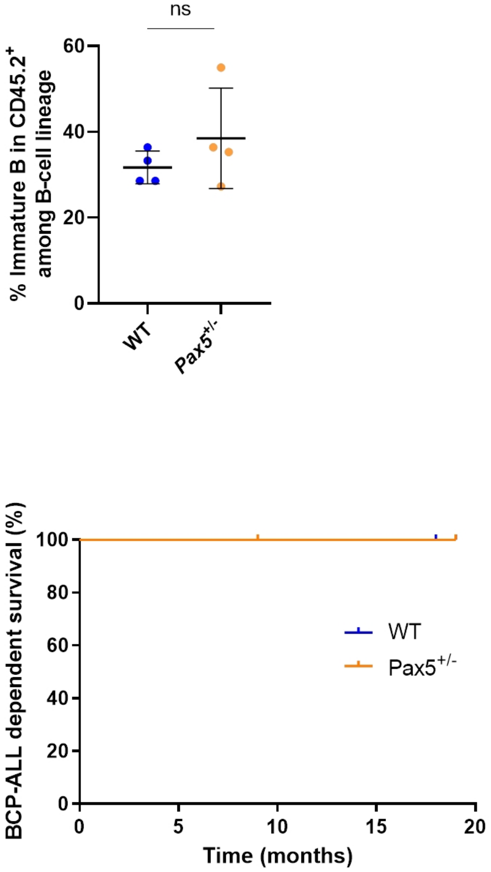

Figure S5:

A: Flow cytometry analysis showing the percentage of engrafted donor CD45.2$^+$ pre-BII cells in lethally irradiated recipients 72h after transplantation in the bone marrow (BM). Donor cells were a pool of n=4, 11 weeks old either *Pax5*$^{+/-}$ or wild-type (WT) mice, each transplanted into n=5 CD45.1$^+$ lethally irradiated recipients. No support whole bone marrow cells (WBMCs) were used for this experimental setup. Displayed is the percentage of IgM$^+$ (Immature) cells that matured from transplanted pre-BII cells among the B-cell lineage (CD19$^+$ B220$^+$). Individual values with mean and SD. A two-tailed Student's unpaired t-test was performed for the statistical analysis. ns = not significant.

B: Long term cohort showing no B-cell precursor acute lymphoblastic leukemia (BCP-ALL) development in CD45.1 recipient mice (n=5 receiving *Pax5*$^{+/-}$ and n=5 receiving WT cells) transplanted each with sorted 1x10^5 pre-BII cells mixed with 5x10^5 support CD45.1 WBMCs (pool of n=4 donor mice, all 11 weeks old for each genotype) and monitored for up to 20 months of age.

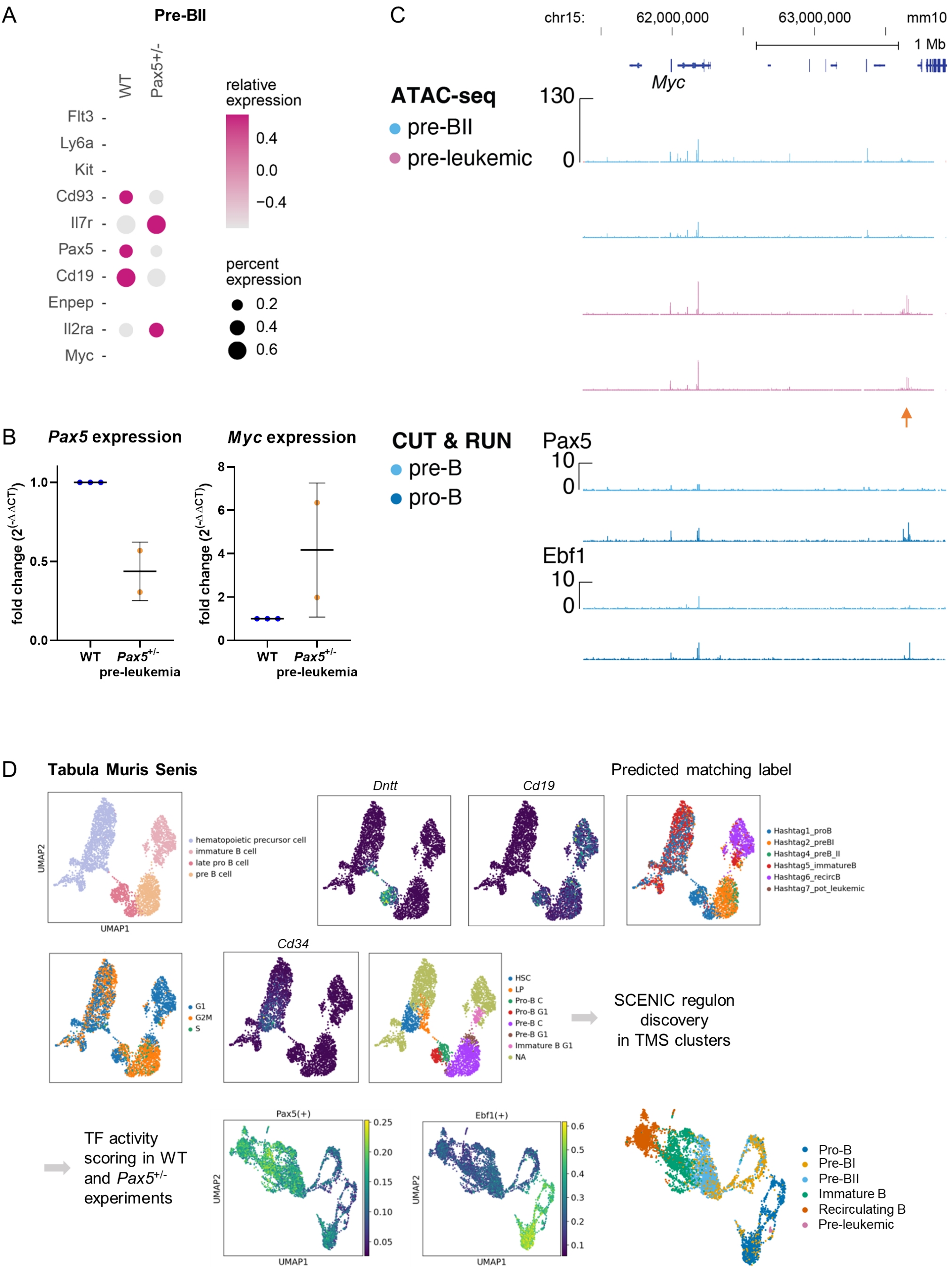

A
Pre-BII
WT
Pax5+/-
Flt3
Ly6a
Kit
Cd93
Il7r
Pax5
Cd19
Enpep
Il2ra
Myc
relative expression
0.4
0.0
−0.4
percent expression
0.2
0.4
0.6
B
Pax5 expression
Myc expression
fold change (2(-ΔΔCT))
WT
Pax5+/- pre-leukemia
C
chr15:
62,000,000
63,000,000
mm10
1 Mb
Myc
ATAC-seq
pre-BII
pre-leukemic
130
0
CUT & RUN
pre-B
pro-B
Pax5
10
0
Ebf1
10
0
D
Tabula Muris Senis
hematopoietic precursor cell
immature B cell
late pro B cell
pre B cell
UMAP1
UMAP2
Dntt
Cd19
Predicted matching label
Hashtag1_proB
Hashtag2_preBI
Hashtag4_preB_II
Hashtag5_immatureB
Hashtag6_recircB
Hashtag7_pot_leukemic
G1
G2M
S
Cd34
HSC
LP
Pro-B C
Pro-B G1
Pre-B C
Pre-B G1
Immature B G1
NA
SCENIC regulon discovery in TMS clusters
TF activity scoring in WT and Pax5+/- experiments
Pax5(+)
Ebf1(+)
Pro-B
Pre-BI
Pre-BII
Immature B
Recirculating B
Pre-leukemic

E

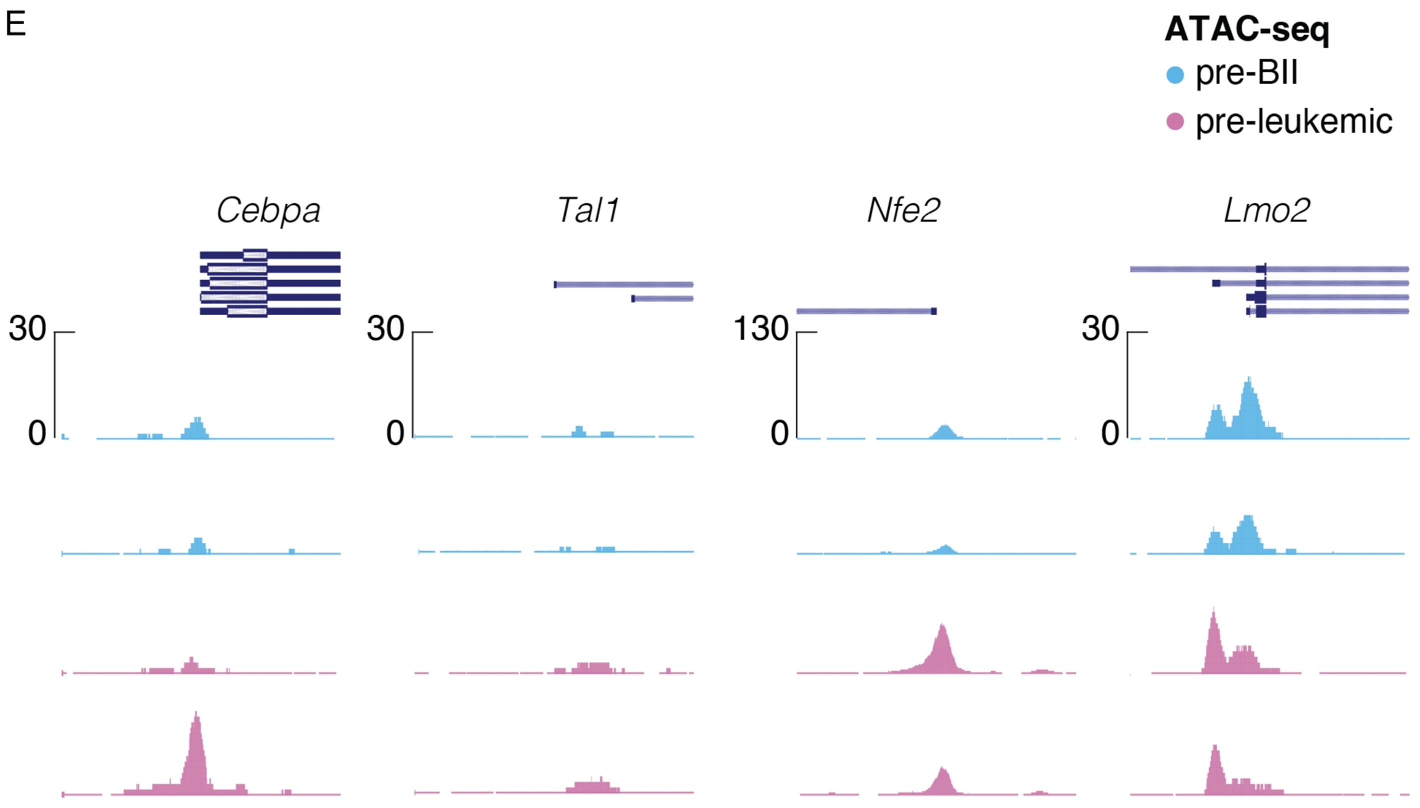
ATAC-seq
pre-BII
pre-leukemic
Cebpa
Tal1
Nfe2
Lmo2
30
0
30
0
130
0
30
0

F

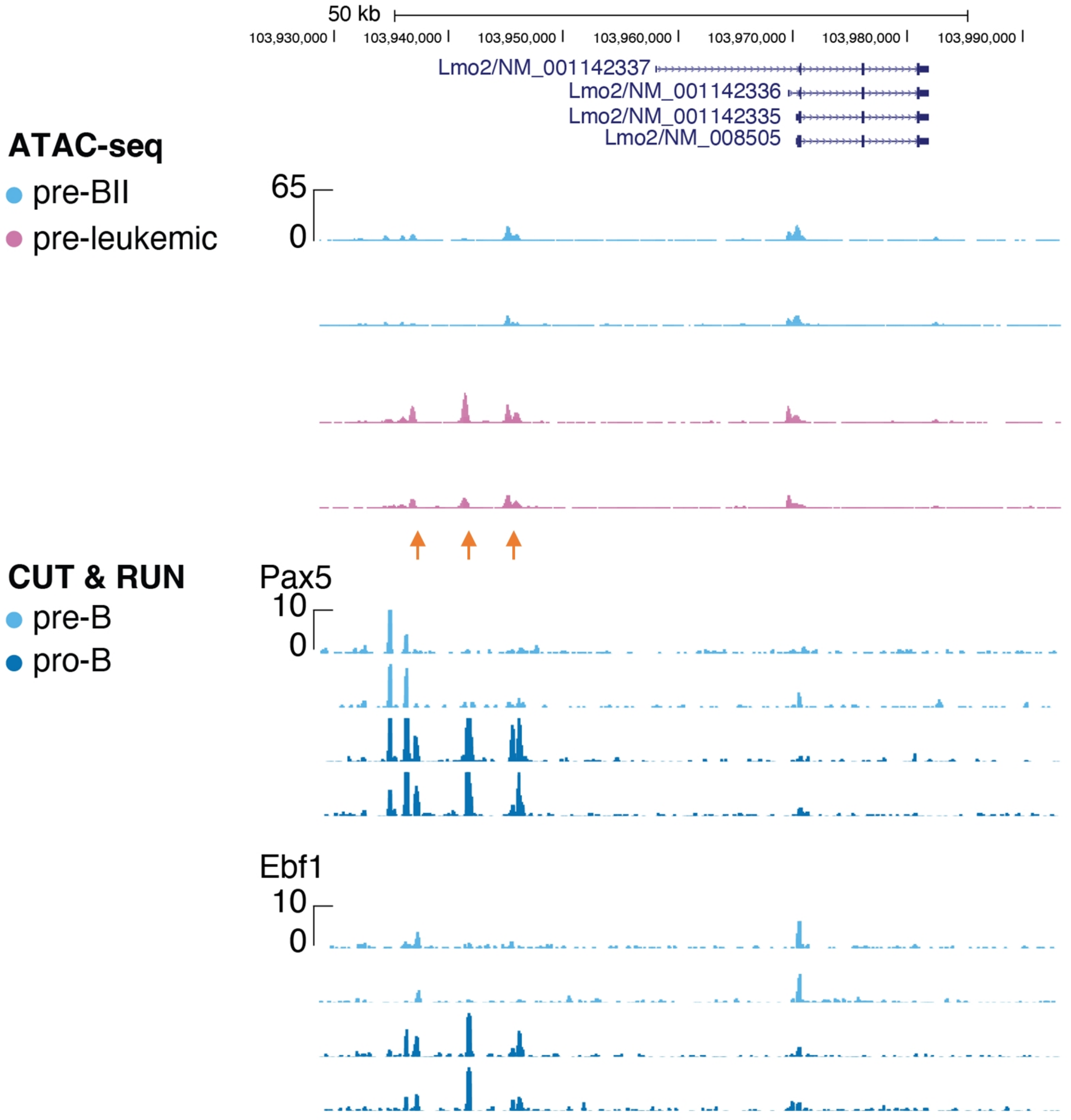
50 kb
103,930,000
103,940,000
103,950,000
103,960,000
103,970,000
103,980,000
103,990,000
Lmo2/NM_001142337
Lmo2/NM_001142336
Lmo2/NM_001142335
Lmo2/NM_008505
ATAC-seq
pre-BII
pre-leukemic
65
0
CUT & RUN
pre-B
pro-B
Pax5
10
0
Ebf1
10
0

Figure S6:

A: Gene expression profile of selected genes in *Pax5*$^{+/-}$ pre-BII cells compared to wild-type (WT) pre-BII cells extracted from the single-cell RNA-Sequencing analysis as a dot plot heatmap.

B: qRT-PCR analysis showing *Pax5* (left) and *Myc* (right) gene expression in B220-enriched bone marrow cells of two *Pax5*$^{+/-}$ mice with a detectable pre-leukemic population (n=2, pre-leukemia 5 and 7) compared to pro-B cells of WT animals (n=3). Displayed are individual values with mean and SD.

C: Chromatin accessibility and transcription factor (TF) binding at *Myc* locus (mm10, chr15:61,369,121-63,923,764). Upper Panel: The WT pre-BII and *Pax5*$^{+/-}$ pre-leukemic ATAC-seq signal tracks show differential activity at the distal enhancer (BENC, indicated by the arrow) that regulates *Myc* expression in B cell precursors (*Somasundaram et al.*). Lower Panel: CUT & RUN data from WT pre-B and pro-B cells displaying co-localization of this distal enhancer BENC region with binding sites for PAX5 and EBF1 (retrieved and reanalyzed from *Fedl et al.*).

D: Reference bone marrow (BM) data from Tabula Muris Senis (TMS). Cells corresponding to clusters annotated on the UMAP were compared to those isolated by flow cytometry in this study based on marker genes (middle) and label transfer analysis (on the right). Further sub-clustering shown below was used to define cell groups for TF regulon discovery. The respective regulons were used to score single cell profiles generated in this study.

E: Chromatin accessibility at TF gene loci. The WT pre-BII and *Pax5*$^{+/-}$ pre-leukemic (pre-leukemia 5 and 7) ATAC-seq signal tracks are shown from a 5 kb region centered at the transcriptional start site (TSS) (*Cebpa*: chr7:35,116,803-35,121,803, *Tal1*: chr4:115053926-115058926, *Nfe2*: chr15:103255903-103260903, *Lmo2*: chr2:103967787-103973787).

F: Chromatin accessibility and TF binding at *Lmo2* locus (chr2:103,880,253-103,993,310) is shown as in C). Three distal enhancers that gain chromatin access and that are bound by PAX5 and EBF1 more strongly in pro-B cells are indicated by arrows.

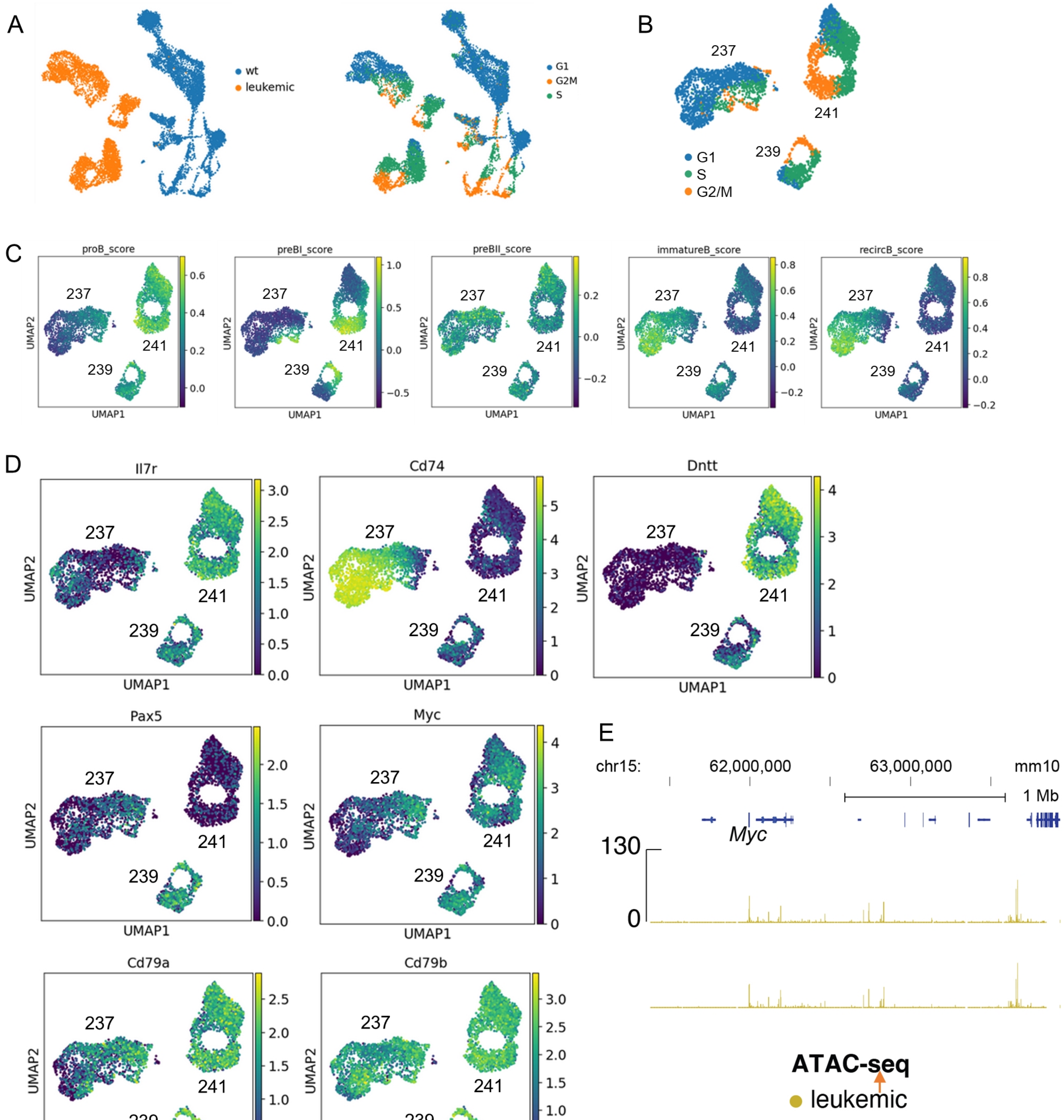

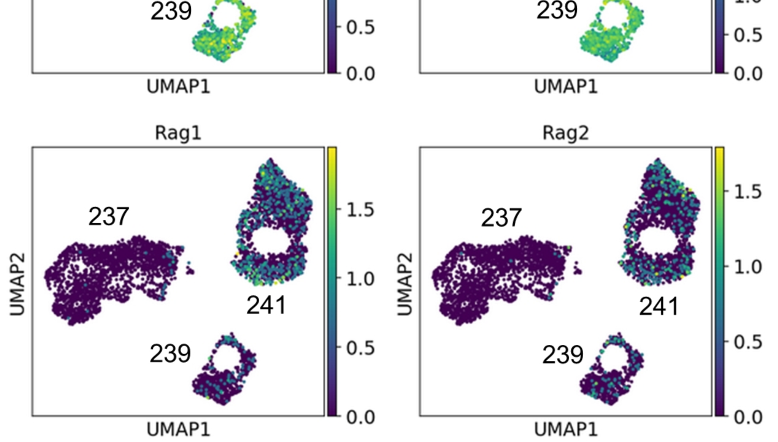

Figure S7:

A: Single-cell (sc) RNA-Sequencing analysis showing differential clustering between leukemic and wild-type (WT) cells (left) is shown on the UMAP. The respective cell-cycle state is displayed on the right.

B: Cell-cycle state of *Pax5*$^{+/-}$ B-cell precursor acute lymphoblastic leukemias (BCP-ALLs) annotated at sc level on the UMAP.

C: Presentation of the *Pax5*$^{+/-}$ leukemias according to the annotated differentiation stage.

D: Gene expression of selected genes for the *Pax5*$^{+/-}$ leukemias.

E: Chromatin accessibility at Myc locus in *Pax5*$^{+/-}$ BCP-ALL 241 (mm10, chr15:61,369,121-63,923,764, the same region as in Supplementary Figure 6C). The arrow indicates additional enhancers that gain chromatin access.
